# The first chromosome-level cubozoan genome differentiates unique and common features in different cnidarian lineages

**DOI:** 10.1186/s12915-026-02579-7

**Published:** 2026-03-24

**Authors:** Sean T. S. Law, Yiqian Li, Chade Li, Wenyan Nong, Kam Ling Chan, Carmen Or, Jian-Wen Qiu, Jerome H. L. Hui

**Affiliations:** 1 School of Life Sciences, Institute of Environment, Energy and Sustainability, State Key Laboratory of Agrobiotechnology, Simon F.S. Li Marine Science Laboratory, The Chinese University of Hong Kong, Hong Kong SAR, China; 2 WWF-Hong Kong, Hong Kong SAR, China; 3 Department of Biology, Hong Kong Baptist University, Hong Kong SAR, China

**Keywords:** Box jellyfish, Sesquiterpenoid hormones, Neuropeptides, microRNAs

## Abstract

**Background:**

Cnidarians are aquatic invertebrates that can be found in both freshwater and marine habitats, including the corals, sea anemones, hydroids, true jellyfish (scyphozoans), and box jellyfish (cubozoans). Despite most cnidarian groups already having representative genomic resources, a high-quality genome of the cubozoan remains lacking.

**Results:**

Here, we obtained the first chromosomal-level genome of the box jellyfish *Tripedalia maipoensis*, having a genome assembly size of 637.8 Mb and a scaffold N50 of 47.1 Mb. By comparing the cubozoan genome to other cnidarian lineages, unique and common features of cnidarians, including cnidarian-specific opsins, toxins, neuropeptides, sesquiterpenoid hormones, and microRNAs, were revealed.

**Conclusions:**

The high-quality genome of a cubozoan presented in this study reveals distinct cnidarian features and provides a crucial missing foundation for further understanding cnidarian evolution more broadly.

**Supplementary Information:**

The online version contains supplementary material available at 10.1186/s12915-026-02579-7.

## Background

The Cnidaria is a diverse phylum of invertebrates characterized by unique stinging cells used for predation and defense. It is estimated that approximately 10,000 species of cnidarians inhabit freshwater and marine environments worldwide, playing important roles in the ecosystem and human society. Cnidarians are broadly divided into several major groups, including Anthozoa (corals and sea anemones), Cubozoa (box jellyfishes), Hydrozoa (hydroids), Myxozoa, Scyphozoa (true jellyfishes), and Staurozoa (stalked jellyfishes).

**Fig. 1:**
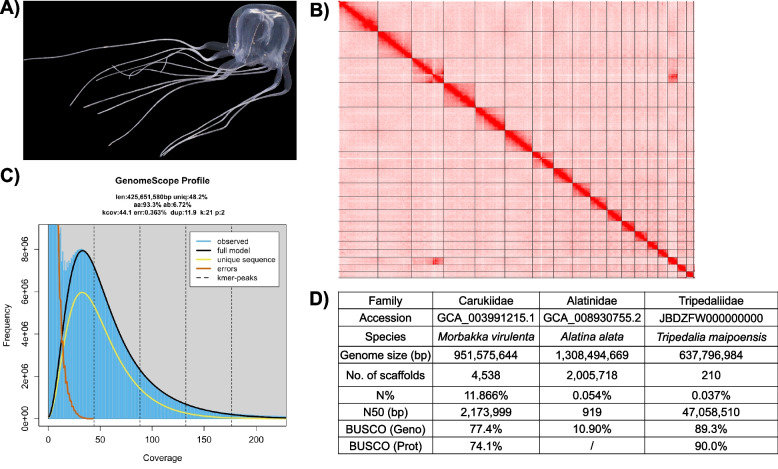
**A** Photo of box jellyfish *Tripedalia maipoensis*. **B** Omni-C contact map of the genome assembly. **C** GenomeScope plot. **D** Statistics of box jellyfish genomes in previous studies and this study

Genomics has emerged as a critical field for unraveling the biology and evolution of various animals. With advancements in sequencing technologies, a wealth of genomic resources has been amassed over the last decade for different cnidarian taxa, including the corals [1–3], sea anemones [4–7], hydroids [8, 9], myxozoans [10, 11], true jellyfishes/scyphozoans[12], and stalked jellyfishes/staurozoans [13]. However, genomic resources for box jellyfishes (Cubozoa) remain limited and are primarily available at draft-quality level [14, 15] (Fig. 1D), which hinders our understanding of their evolutionary history.

Cubozoa includes approximately 50 described species of box jellyfish species, which can be further divided into two orders: Carybdeida (characterized by one tentacle per pedalium) and Chirodropida (characterized by multiple tentacles per pedalium) [16]. Box jellyfishes are notable for their potent venom; they are primarily found in the Indo-Pacific and Atlantic Oceans [17]. *Tripedalia maipoensis* is a recently described new box jellyfish species in the Tripedaliidae family [18], which possess three pedalia at each bell corner, a tentacle on each Pedalium, and forked canals on the velarium [18]. In this study, we present a high-quality genome of a box jellyfish *T. maipoensis* and compare it with other published cnidarian genomes, including macrosynteny, gene families of cnidopsins, toxins, neuropeptides, sesquiterpenoid hormones, and microRNAs, offering novel insights into the genomic evolution of the Cnidaria.

## Results and discussion

### The first high-quality cubozoan genome

Here, we present the first high-quality genome assembly of *T. maipoensis*, which is 637.8 Mb in size with sequence continuity of scaffold N50 = 47.1 Mb and L90 = 16, and 96.76% of the sequences are anchored to 18 chromosomes scaffolded by YaHS [19] version 1.2a.2 (Fig. 1A–B; Additional File 1: Table S1–S3). The mean depth of Omni-C data coverage was similar between most of these 18 scaffolds, with a lower mean depth observed in a region of scaffold 3 (Additional File 2: Fig. S1). In addition, substantial contact was observed between scaffolds 3 and 16 (Fig. 1B). We further scaffolded another version (v2) of genome assembly with YaHS [19] version 1.2.2, which joined scaffolds 3 and 16 of the first assembly (v1) as scaffold 1 (Additional File 2: Fig. S2 and S3). However, the contact map of scaffold 1 in v2 assembly reflected potential misjoints and weak signals of interaction in partial intra-chromosomal regions (Additional File 2: Fig. S2). Therefore, we used v1 assembly for further genomic analyses. The assembled genome size is slightly larger than the genome size estimation by GenomeScope and is comparable to the other published cubozoan genomes (Fig. 1C–D; Additional File 1: Table S3). Transposable elements (TEs) account for 34.56% of the genome, about half of which (~ 15.78% of the genome) is contributed by LINEs with recent expansion (Additional file 2: Fig. S4). Utilising the Benchmarking Universal Single-Copy Orthologs (BUSCO v5.5.0, metazoa_odb10) as an estimation of completeness, 82.6% and 90.0% were respectively found on the genome and predicted gene models (Fig. 1D; Additional File 1: Table S3). The BUSCO score of the predicted gene model is comparable to the genome-guided transcriptome assembly (94.2%), indicating a high level of completeness of the predicted gene model set (Additional File 1: Table S3).

**Fig. 2:**
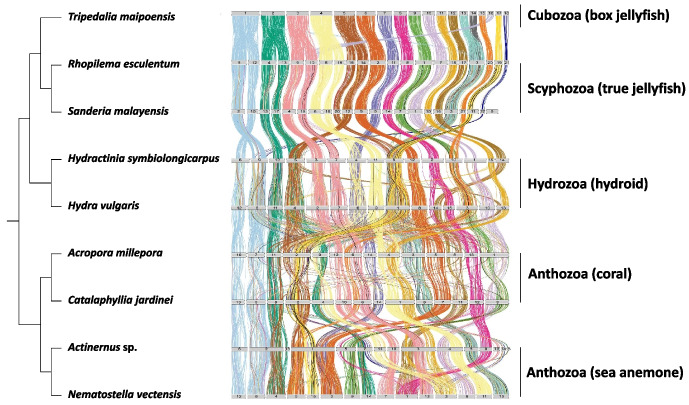
Macrosynteny between genomes of *T. maipoensis*, eight other cnidarians, and a ctenophore. The coloured lines represent connected orthologous genes from the 18 respective chromosomes of *T*. *maipoensis*

Macrosynteny analyses further revealed *T. maipoensis* shares 1-to-1 syntenic blocks with other chromosomal-level cnidarian genomes, with a higher number of homologous chromosomes and a lower macrosynteny mixing score compared to the scyphozoan genomes than to the anthozoan and hydrozoan genomes (Fig. 2, Additional File 2: Fig. S5 and S6). Notably, the longest 5 chromosomes (chr 1–5) of *T. maipoensis* are derived from fusion events and are homologous to two chromosomes in scyphozoans and hydrozoans. In addition, 1-to-1 syntenic blocks were also identified between the genomes of *T. maipoensis* and *Morbakka virulenta* (Additional File 2: Fig. S7).

### Cnidopsin and toxin gene gains in cubozoans

**Fig. 3:**
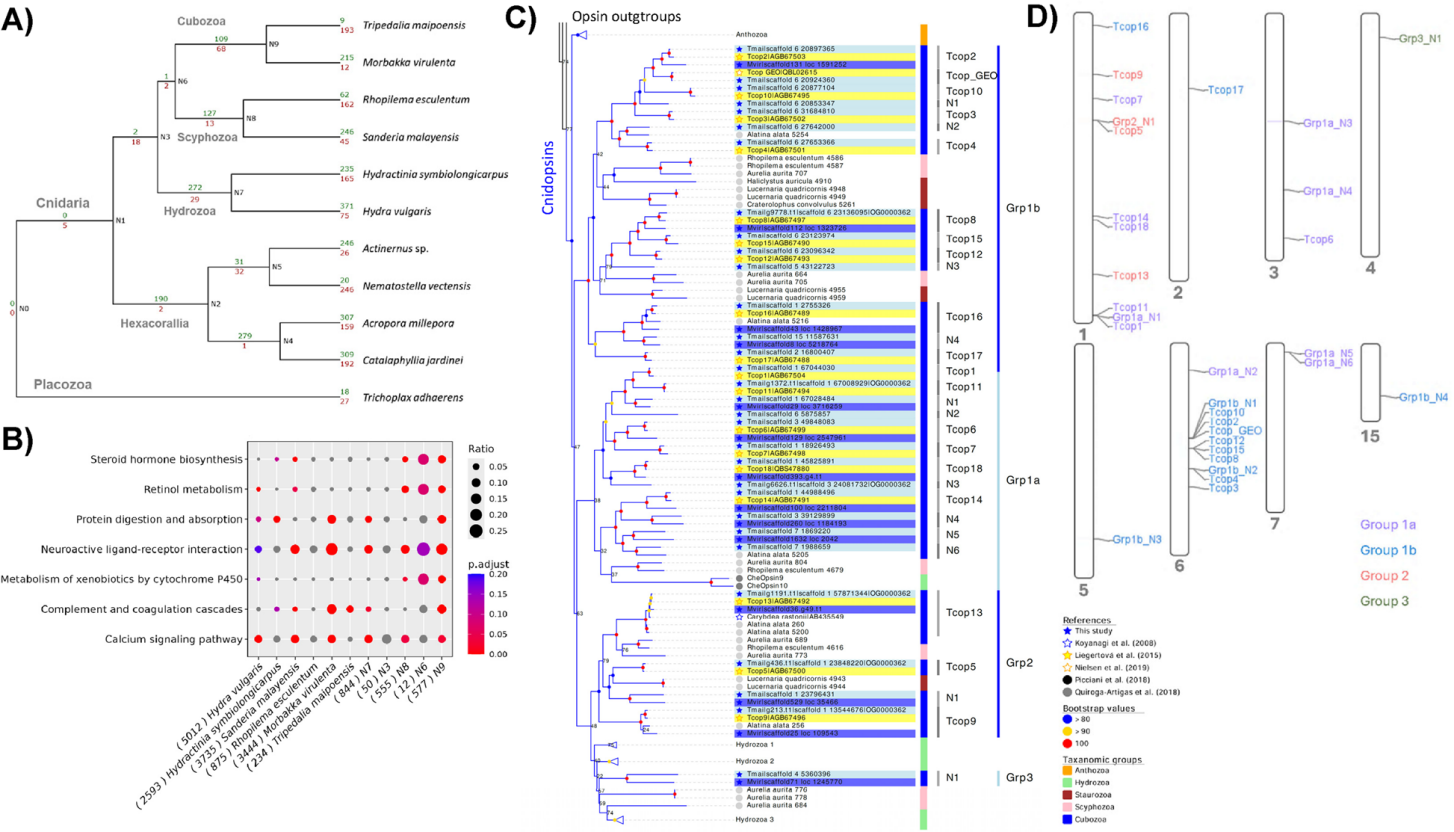
**A** Gene family expansion and contraction of the orthologous groups are indicated in green and red, respectively. **B** Enriched KEGG pathways of gene family gains. The gene ratio indicates the number of gene gains divided by the total number of genes in the respective gene pathway. **C** Maximum-likelihood tree of cnidopsins genes. Cnidopsins identified from *T. maipoensis* and *Morbakka virulenta* are highlighted in lightblue and blue, respectively, while the reference sequences from *Tripedalia cystophora* are highlighted in yellow. The internal nodes of opsins other than cnidopsins are collapsed for better visualisation (see Additional File 2: Fig. S9 for the full tree). **D** Gene locations of 31 cnidopsins in the genome of *T. maipoensis*. Group 1a, 1b, 2 and 3 cnidopsins were labelled in purple, blue and orange and green, respectively

To understand the patterns of gene gains in the cubozoan lineage, we compared predicted genes across ten cnidarian genomes and two non-cnidarians (Fig. 3A; Additional File 1: Table S4). Gene enrichment analyses identified gene gains in different KEGG pathways in *T. maipoensis* and node “N9” in Fig. 3A, including retinol metabolism (ko00830), steroid hormone biosynthesis (ko00140), metabolism of xenobiotics by cytochrome P450 (ko00980), serotonergic synapse (ko04726), and neuroactive ligand-receptor interaction (ko04080) (Fig. 3B, Additional File 1: Table S5). Specifically, we found that gene families including cytochrome P450 (Pfam00067), UDP-glucuronosyltransferase (Pfam00201), trypsin (Pfam00089) and G protein-coupled receptors (Pfam00001) were expanded in the cubozoan lineage (Additional File 2: Fig. S8).

Contrary to hydrozoans and scyphozoans, cubozoans possess a special visual system that involves four different eye types located in each of the four rhopalia, namely one upper lens-eye, one lower lens-eye, two pit eyes, and two slit eyes, adding up to a total set of 24 eyes [18, 20, 21]. Previous studies have also found that certain opsin gene family members are specific to cnidarians, namely cnidopsins [22–25], including 19 cnidopsins that have been identified from the transcriptomes of box jellyfish *Tripedalia cystophora* (Tcop1-18 in [23] and *TcGEO* in [26]). Using sequence homology searches, we have identified a total of 108 and 123 opsin gene candidates in *T. maipoensis* and *Morbakka virulenta* genomes, respectively. Phylogenetic analyses further confirmed that 31 and 14 cnidarian-specific opsins could be respectively identified in *T. maipoensis* and *M. virulenta* (Fig. 3C–D; Additional File 1: Table S6; Additional File 2: Fig. S9–S12). Most of the cnidarian-specific opsins in *T. maipoensis* (*n* = 30) and *M. virulenta* were clustered into clades of group 1a, 1b and 2 as previously reported [23]. In addition, we also revealed that one cnidopsin each from *T. maipoensis* and *M. virulenta* were clustered with the Hydrozoan clade, which we termed as group 3 in this study. High level of sequence conservation in functional sites could also be observed between most cubozoan cnidopsin orthologues, including the lysine residue (K296) for chromophore binding and the cysteine pair (C110 and C187) for the disulphide bridge that stabilises the opsin structure [27, 28] (Additional File 2: Fig. S13). In sum, the first set of cubozoan cnidopsins revealed in this study could provide a valuable baseline for further understanding the functions of different cnidopsins.

**Fig. 4:**
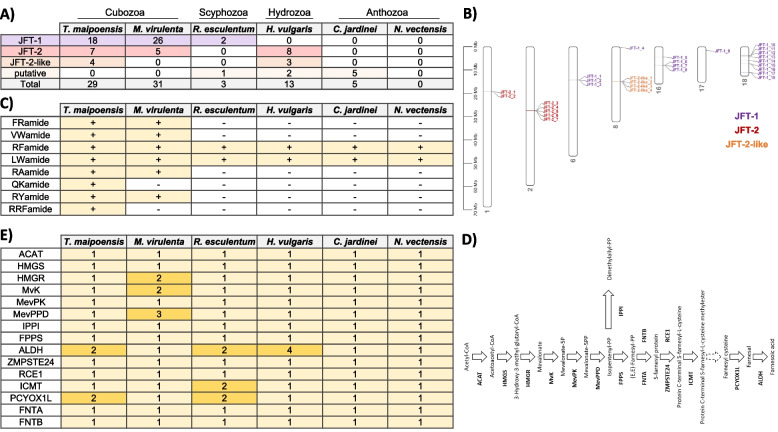
**A** Jellyfish toxins (JFTs) in cnidarians. **B** Locations of JFTs in *T. maipoensis* genome. **C** Neuropeptide precursor genes in cnidarians. The presence and absence of the neuropeptide precursor genes are indicated with “ + ” and “ − ”, respectively. **D** Schematic diagram showing the biosynthetic pathway of sesquiterpenoid hormones. Abbreviation: ACAT: acetyl-CoA acetyltransferase; HMGS: 3-hydroxy-3-methylglutaryl-CoA synthase; HMGR: 3-hydroxy-3-methylglutaryl-CoA reductase; MvK: mevalonate kinase; MevPK: phosphomevalonate kinase; MevPPD: mevalonate (Diphospho) decarboxylase; FPPS: farnesyl diphosphate synthase; IPPI: isopentenyl-diphosphate delta isomerase; FNTA: farnesyltransferase, CAAX box, alpha; FNTB: farnesyltransferase, CAAX box, beta; ZMPSTE24: STE24 endopeptidase; RCE1: prenyl protein protease; ICMT: isoprenylcysteine carboxyl methyltransferase; PCYOX1L: prenylcysteine oxidase 1; ALDH: aldehyde dehydrogenase III. **E** Gene copy numbers of sesquiterpenoid biosynthetic pathway genes identified in respective cnidarian genomes

The cubozoan or box jellyfish are also well known for their potent and rapid-acting venom, and previous studies have suggested that jellyfish toxins (JFTs) are cnidarian-specific pore-forming toxins that contribute to the potency of cubozoan venoms [29–31]. In the genomes of *T. maipoensis* and *M. virulenta*, 29 and 31 JFTs were identified, respectively (Fig. 4A; Additional File 1: Table S11). Phylogenetic analysis with JFTs [29] revealed that a greater number of gene copies in the JFT-1 clade (*n* = 18 in *T. maipoensis* and *n* = 26 in *M. virulenta*) than the JFT-2 clade (*n* = 7 in *T. maipoensis* and *n* = 5 in *M. virulenta*) (Additional File 2: Fig. S14). Many of the JFT genes are genomically located next to each other, suggesting that gene duplication events occurred in these lineages (24 in *T. maipoensis* and 22 in *M. virulenta*) (Fig. 4B; Additional File 1: Table S11). Synteny analyses between *T. maipoensis* and *M. virulenta* could not identify any annotated JFT genes in the 301 syntenic blocks. Thus, whether these JFT gene duplicates were derived from an ancestral process of tandem duplication or occurred independently warrants further investigation.

### Conservation of neuropeptides and sesquiterpenoid farnesoic acid hormones in cnidarians

Neuropeptides are important signaling molecules in the nervous system that regulate various physiological activities in animals. A previous study has shown that neuropeptides in cnidarians and bilaterians do not share structural similarities [32]. In *T. maipoensis*, nine neuropeptide genes including FRamide, VWamide, RFamide, LWamide, RAamide, OKamide, RRFamide, and RYamide could be identified, and in agreement with previous studies [26] (Fig. 4C; Additional File 1: Table S8; Additional File 2: Fig. S15).

The sesquiterpenoid hormones are well known to regulate the development and reproduction in insects and are now found in different invertebrates, including cnidarians [6, 12, 33]. In *T. maipoensis*, all genes involved in sesquiterpenoid hormone biosynthetic pathways can be identified for producing farnesoic acid (Fig. 4D–E; Additional File 1: Table S9). These genes can also be identified in other investigated cnidarian lineages including scyphozoans [12], anthozoans [2], and hydrozoan [33], suggesting that the production of sesquiterpenoid hormone farnesoic acid is a conserved feature throughout cnidarians. These studies show that cnidarians shared similar sesquiterpenoid farneosoic acid hormone but different neuropeptides to those in the bilaterians.

### Conserved microRNAs between cnidarians but not to bilaterians

**Fig. 5:**
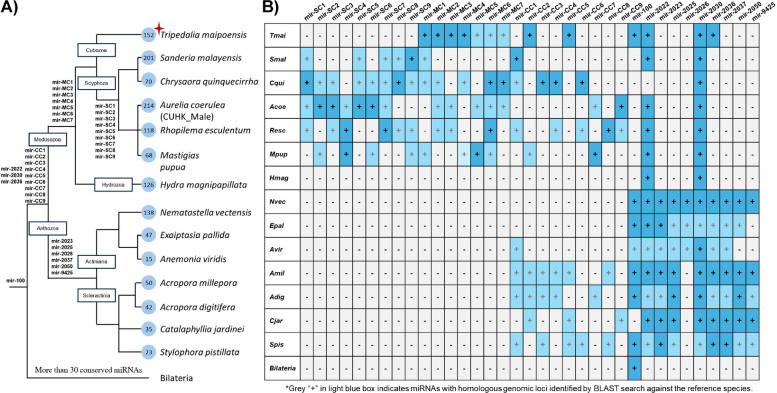
**A** Evolutionary gains of microRNAs in cnidarians. The numbers within the circles at the end of the branches represent the total number of identified microRNAs in each species. The phylogenetic topology was adopted from previous cnidarian studies [2, 12, 39]. **B** Conservation of microRNAs shared at least in three species. “ + ” in the blue box imply the presence of microRNAs, whereas “ − ” in grey box show the absence of microRNAs in this species. *Tmai*, *Tripedalia maipoensis*; *Smal, Sanderia malayensis*; *Cqui*, *Chrysaora quinquecirrha*; *Acoe*, *Aurelia coerulea*; *Resc*, *Rhopilema esculentum*; *Mpap*, *Mastigias papua*; *Hmal*, *Hydra magnipapillata*; *Nvec*, *Nematostella vectensis*; *Epal*, *Exaiptasia pallida*; *Avir, Anemonia viridis*; *Amil*, *Acropora millepora*; *Adig*, *Acropora digitifera*; *Cjar*, *Catalaphyllia jardinei*; *Spis*, *Stylophora pistillata*

MicroRNAs act as crucial post-transcriptional regulators in animals, and they show similarities and differences between cnidarians and bilaterians [34]. For instance, the targeting properties and regulation between microRNAs of cnidarians and bilaterians differ, and there is only one shared microRNA miR-100 between cnidarians and bilaterians [35–38]. Previous studies have identified lineage-specific microRNAs shared among scyphozoans and among anthozoans [6, 12, 39]. By sequencing the small RNAs in *T. maipoensis* and various scyphozoans, we identify a total of 152, 201, 70, 214, 118, and 68 microRNAs in the cubozoan *T. maipoensis* and scyphozoans *Sanderia malayensis*, *Chrysaora quinquecirrha*, *Aurelia coerulea*, *Rhopilema esculentum*, and *Mastigias papua*, respectively (Fig. 5A; Additional File 1: Table S10–S15). Similar to previous studies, miR-100, miR-2022, and miR-2030 could be identified in most cnidarian genomes, including the cubozoan [12, 36, 40]. Nevertheless, miR-2036 which was previously thought to be an anthozoan-specific microRNA, can now also be identified in the cubozoan lineage (Fig. 5B, Additional File 2: Fig. S16). Furthermore, we have identified nine novel microRNAs (namely miR-CC1 to CC9) that exhibit sequence conservation across cubozoan, scyphozoans, and anthozoans; seven novel microRNAs (namely miR-MC1 to MC7) to be conserved between cubozoan and scyphozoans; and nine novel microRNAs (namely miR-SC1 to SC9) to be conserved only in scyphozoans (Fig. 5B, Additional File 2: Fig. S17–S19). Notably, miR-MC6 exhibits sequence homology with the previously reported anthozoan-specific miR-2023, despite a nucleotide mismatch occurring at position 2 of the 3p arm (Additional File 2: Fig. S20). BLASTn search of all identified cnidarian microRNAs against miRbase [41] showed that only miR-100 was shared by cnidarian and bilaterian lineages as previously reported [35, 36]. These findings suggest that there are indeed deeply conserved microRNAs between different cnidarian lineages, despite only one microRNA shared between cnidarians and bilaterians.

## Conclusions

In summary, this study presents the first high-quality genome of a cubozoan, providing a key genomic resource for comparative analyses and filling a crucial gap in our understanding of cnidarian genome evolution. Our findings highlight genomic features that are uniquely acquired in cubozoans, such as cnidopsins and jellyfish toxins, as well as conserved features shared among cnidarians, including the sesquiterpenoid hormone farnesoic acid and various neuropeptides. Additionally, we identify divergences between cnidarians and bilaterians, particularly in the realm of microRNAs.

## Methods

### High molecular weight DNA extraction

*T. maipoensis* individuals were collected in brackish water ponds in Mai Po and were stored in − 80 °C once transported to the laboratory. High molecular weight (HMW) genomic DNA was extracted from ~ 500 mg tissue of a mature *T. maipoensis* medusa individual using CTAB [42] with the modification of adding 1/3 volume 3 M potassium acetate (Buffer P3, Qiagen) to the supernatant after the first chloroform wash. The isolated HMW DNA was subject to quality assessment using the NanoDrop™ One Microvolume UV–Vis Spectrophotometer (Thermo Scientific), Qubit Fluorometer, and overnight pulse-field gel electrophoresis.

### PacBio library preparation and long read sequencing

DNA shearing was carried out using 5.2 µg HMW DNA in 120 µL elution buffer in a g-tube (Covaris) for 6 passes of centrifugation at 2000 × g for 2 min, followed by a DNA purification step using SMRTbell® cleanup beads (PacBio). A SMRTbell library was subsequently prepared using the SMRTbell® prep kit 3.0 (PacBio) following the manufacturer’s instructions. Two microliter of the resulting library was subject to quality assessment using Qubit Fluorometer and pulse-field gel electrophoresis. The final library preparation for sequencing was carried out with Sequel® II binding kit 3.2 (PacBio), which allows SMRT bell structures to be annealed with Sequel II® primer 3.2 and bound with Sequel II® DNA polymerase 2.2 with the addition of Sequel II® DNA Internal Control Complex. The final library was sequenced on the Pacific Biosciences SEQUEL IIe System with the diffusion loading mode set at an on-plate concentration of 90 pM for 30-h movies with 120 min pre-extension. Two SMRT cells were used for the generation of HiFi reads (Additional File 1: Table S1).

### Omni-C library preparation and sequencing

A total of ~ 100 mg tissue of the same individual used in HMW DNA extraction was used for Omni-C library construction using the Dovetail® Omni-C® Library Preparation Kit (Cantata Bio) following the manufacturer’s protocol. The concentration and fragment size of the lysate in the intermediate step and the final library were assessed by Qubit Fluorometer and TapeStation D5000 HS ScreenTape (Agilent), respectively. The resulting library was sent to Novogene (Hong Kong) and sequenced on an Illumina HiSeq-PE150 platform (Additional File 1: Table S1).

### RNA extraction, transcriptome and small RNA sequencing

RNA from an 8-arm juvenile and a mature medusa individual of *T. maipoensis* (different from the individual used for PacBio and Omni-C library sequencing); the arm, bell, tentacle and gonad tissues of *Chrysaora quinquecirrha*; and the bell of *Mastigias papua* were extracted using the mirVana miRNA Isolation Kit (Ambion) according to the manufacturer’s instructions. For *Mastigias papua*, the tissues were pre-treated with an additional step using a CTAB solution (1 M Tris–HCl, 0.2 M EDTA, 5 M NaCl, CTAB, 1% PVP, and 1% beta-mercaptoethanol) for removal of insoluble materials from the samples. The isolated RNA was subject to quality control with gel electrophoresis and NanoDrop™ One Microvolume UV–Vis Spectrophotometer (Thermo Scientific). RNA samples were sent to Novogene (Hong Kong) for total RNA transcriptome library construction and sequencing on the Illumina Novaseq PE150 platform, and for small RNA library construction and sequencing on the Illumina Novaseq 50SE platform (Additional File 1: Table S1).

### Genome assembly and Gene model prediction

De novo genome assembly of *T. maipoensis* was first performed using Hifiasm (version 0.19.8-r603) [43] and then searched against the NT database using blastn (version 2.15.0 +) [44] to remove possible contamination using BlobTools (version 1.1.1)[45]. Subsequently, haplotypic duplications were removed according to the depth of the HiFi reads using “purge_dups” (version 0.0.3) [46]. Proximity ligation data from Omni-C were used to scaffold the assembly using two versions of YaHS [19], version 1.2a.2 and version 1.2.2 with default parameters, respectively, followed by manual checking using Juicebox (version 1.11.08) [47]. Briefly, Omni-C reads were mapped and aligned by BWA (version 0.7.18-r1243-dirty) [48] with parameters “mem -5SP -T0”, the parsing module of the pairtools pipeline [49] was used to find ligation junctions with parameters “–min-mapq 40 –walks-policy 5unique –max-inter-align-gap 30 –nproc-in 8 –nproc-out 8”. The parsed pairs were then sorted using pairtools sort under default parameters. PCR duplicate pairs were removed using pairtools dedup with parameters “–nproc-in 8 –nproc-out 8 –mark-dups”. The pairs file was split using pairtools split with default parameters and used to generate the contact matrix using juicertools and Juicebox. The scaffolding results of the two genome assemblies versions (v1 for YaHS version 1.2a.2 and v2 for YaHS version 1.2.2) were compared with genome alignments using MashMap [50] with parameters of filter mode and alignment percentage identity set as one-to-one and 95% (“–filter_mode one-to-one –perc_identity 95”). For the genomic characteristics of the assembly, the k-mer count and histogram were generated at *k* = 21 from the Omni-C reads using Jellyfish (version 2.3.0)[51] with the parameters “count -C -m 21 -s 1,000,000,000 -t 10”, and the reads.histo was uploaded to GenomeScope to estimate genome heterozygosity, repeat content and size using default parameters (version 2.0) (http://qb.cshl.edu/genomescope/genomescope2.0/) [52]. The resulting GenomeScope plots are shown in Fig. 1C. BUSCO (v5.5.0) [53] analysis was performed using the metazoa_odb10 database to assess the completeness of the final genome assembly. For gene model prediction, the genomes were soft-masked using redmask (version 0.0.2) [54]. RNA sequencing data of the 8-arm juvenile and mature medusa were first processed using Trimmomatic (version 0.39) [55] with parameters “TruSeq3-PE.fa:2:30:10 SLIDINGWINDOW:4:5 LEADING:5 TRAILING:5 MINLEN:25” and kraken2 (v2. 0.8 with kraken2 database k2_standard_20210517) [56], and then aligned to the soft-masked repeat genome using hisat2 (version 2.2.1) [57] to generate the bam file. A total of 429,757 Cnidaria reference protein sequences were downloaded from NCBI on 27 May 2024 as protein hits, along with the RNA bam file, to perform genome annotation using Braker (version 3.0.8) [58] with default parameters. The same pipeline was used to perform genome annotation on the other three cubozoan genomes for comparison purposes. Two RNA-seq datasets (SRR7983770 and SRR7983771) were downloaded from the National Center for Biotechnology Information (NCBI) to provide transcriptome information for the *Morbakka virulenta* genome (GCA_003991215.1), and four RNA-seq datasets (SRR8101947, SRR8101944, SRR8101946 and SRR8101945) were downloaded to provide transcriptome information for the *Chironex yamaguchii* genome (GCA_024741275.1). However, the Braker pipeline could not be run for the *Alatina alata* (GCA_008930755.2), *Carybdea cf. marsupialis* (Linnaeus, 1758) (GCA_010016065.2) and *Tamoya ohboya* (GCA_028566775.1) genomes, as these contain highly fragmented assemblies (see Additional File 1: Table S3). To assess the completeness of the predicted gene models, a genome-guided transcriptome assembly was conducted using Trinity (v2.8.4) [59]. BUSCO (v5.5.0) [53] analysis was then performed with the database metazoa_odb10.

### Repeat annotation

Transposable elements (TEs) were annotated using the Earl Grey (v1.2) [60] pipeline with the option “-r cnidaria” specified for the initial mask of known TEs. A repeat landscape plot that illustrates the divergence between the annotated individual TEs and their respective consensus sequence in Kimura distance was generated automatically in the pipeline.

### Macrosynteny analyses

Macrosynteny analyses were conducted by comparing the *T. maipoensis* genome with other chromosome-level assemblies using SyntenyFinder [61] (Additional File 1: Table S4). In brief, single-copy orthologues were first identified by OrthoFinder v2.5.5 [62] and were then further used to generate a dataset of orthologous genes linked with the genomic position. The gene linkage between each pair of genomes was analyzed in the SyntenyFinder pipeline and visualized using RIdeogram [63]. Furthermore, a synteny mixing score was generated between each genome pair to reflect the degree of chromosomal rearrangement as documented in [61]. A Spearman’s rank correlation coefficient (*ρ*) was generated to quantify the extent of intra-chromosomal gene shuffling, and the synteny mixing score was then calculated by 1 minus the absolute value of Spearman’s coefficient (*ρ*). The Oxford dot plots of the genome pair were also generated as previously described [64] where orthologous genes were determined by mutual best hits using Diamond blastp (v2.1.8) [65] with a cut-off *e*-value of 0.001. The genomic positions and numbers of orthologous genes were then indexed and compared to generate the synteny Oxford dot plot. The orthologous genes were further tested for significance with the one-tailed test for hypergeometric distribution using Fisher’s exact test, and the *p* value was adjusted with the Benjamini–Hochberg method. In addition, syntenic analysis was performed between *T. maipoensis* and *Morbakka virulenta* genomes using GENESPACE [66] with default parameters. In brief, gene orthology was inferred by OrthoFinder [62] and collinearity blocks were detected with a minimum block size of 5 collinear genes using MCScanX [67].

### Gene gain and functional enrichment analyses

Gene orthology was inferred from the longest transcripts of the gene set extracted from 11 genomes, including 10 cnidarian genomes and a placozoan, using OrthoFinder v2.5.5 [62] (Additional File 1: Table S4). The species tree and orthologue assignment generated from OrthoFinder were used as the input for CAFE5 [68]. Functional annotations were performed in each gene set using eggnog [69] for the assignment of annotation terms, including Gene Ontology (GO), Kyoto Encyclopedia of Genes and Genomes (KEGG), and KEGG Orthology (KO). Enrichment analysis on gene gain was performed with the function “compareCluster()” in the *R* package “clusterProfiler” v.3.16.1[70] with *p*-value adjusted using Benjamini and Hochberg (BH) method and *p*-value cutoff at 0.05. The gene family gains were further validated with the construction of a phylogenetic tree for determining if the gene family was expanded in the cubozoan lineage when a conserved domain (pfam) was identified. Protein family candidates were searched in the gene set using HMMER [71] (version 3.3.1; cut-off *e*-value < 10 − 5) and were further screened using Reverse Position-Specific BLAST (RPS-BLAST) against the Conserved Domain Database [72]. The domain sequence was extracted and aligned using MAFFT v7.271 [73]. A phylogenetic tree was constructed for each protein family using FastTree with the default parameters [74], which was then visualized in evolview3 [75].

### Opsin gene family analyses

Putative candidates of opsin genes in the *T. maipoensis* and *Morbakka virulenta* genomes were identified using 18 opsin protein sequences from the cubozoan jellyfish *Tripedalia cystophora* [23] as a query for sequence homology search with tblastn (*e*-value cut-off at 1e-05). Putative opsin candidates were further validated with phylogenetic tree construction. Briefly, the opsin candidates were aligned with other documented opsins in Cnidaria [22, 25, 26, 76, 77] and other non-opsin G-protein coupled receptor sequences as outgroup [77]. The aligned sequences were trimmed as described in [25] using TrimAl [78], which included removal of positions with a gap threshold of 90% and spurious sequences that do not possess an overlap of 50% with other sequences in 65% of their residues. A maximum-likelihood tree was constructed with IQ-TREE2 [79] with 1,000 bootstraps using the LG + F + R10 model as determined by IQ-TREE2, which is also similar to other published studies [25, 77]. The resulting tree was visualised in evolview v3 [75]. Gene positions on chromosomes were visualised with TBtools-II[80]. To reveal the cnidopsin orthology, the trimmed sequence alignment was visualized with Jalview v2 [81]. Conserved structural and functional opsin motifs corresponding to the bovine rhodopsin numbering system were inspected as previously described [77].

### Jellyfish toxin gene identification

Pore-forming jellyfish toxin (JFT) sequences extracted from previous studies [29, 82] were used as a query in tblastn search against the cnidarian (*e*-value cut-off at 1e-05). The JFT gene candidates were aligned with reference sequences together with the bacterial Cry toxins as outgroups as described in [29] using MAFFT v7.271 [73]. The aligned sequences were trimmed with TrimAl [78]. A maximum-likelihood phylogenetic tree was constructed with FastTree with the default parameters [74], followed by visualization in evolview v3 [75]. The locations of *T. maipoensis* JFTs gene were visualized in TBtools-II [80].

### Sesquiterpenoid hormonal pathway genes and neuropeptide gene identification

Sesquiterpenoid hormonal pathway genes of various cnidarians were retrieved from KEGG and previous studies [6] for gene annotation in *T. maipoensis* and *Morbakka virulenta*. Retrieved gene sequences were used as query and carried out blastp (TBtools-II v2.310) [80] and tblastn (*e*-value of 1e-4) to identify potential candidates in predicted gene models and genomes, respectively. Potential candidates were also used to carry out reciprocal blastp in the NCBI ClusteredNR database with default settings. Neuropeptide precursor genes of *Tripedalia cystophora* [26] were retrieved from NCBI as a query for carrying out blastp and tblastn against gene model and assembled genome, respectively, to identify potential neuropeptide precursor gene candidates in different cnidarians with an *e*-value of 1e-4 through TBtools-II (v2.330) [80]. Reciprocal blastp was conducted for potential candidates against the NCBI ClusteredNR database to identify confident candidates with default settings. Identified neuropeptides were further submitted to the DeepNeuroPred function of NeuroPep2.0 [83] to predict signal sequences and cleavage sites.

### microRNA analyses

For the small RNA data in *Tripedalia maipoensis*, *Chrysaora quinquecirrha*, *Mastigias papua*, *Aurelia coerulea*, *Sanderia malayensis*, and *Rhopilema esculentum*, small RNA sequencing reads with adaptor sequences were trimmed, and Phred quality scores of less than 20 were deleted. Processed readings ranging in size from 18 to 27 bp were then mapped to their respective genomes using mapper.pl module of the mirDeep2 package [84]. miRDeep2 was used to identify novel microRNAs, which were then manually checked to ensure they fulfilled the criteria of MirGeneDB (http://mirgenedb.org/information) [85]. The main criteria of MirgeneDB for determining authentic miRNAs include: the abundance of sequencing reads (both 5p and 3p reads should have expression); and base pairs in at least 16 of the ~ 22 nucleotides; the 5p and 3p reads are offset by two nucleotides; the length of the loop is at least eight nucleotides long. Small RNA sequencing data from each species were examined independently for the presence of novel microRNAs. Only candidates fulfilling all MirGeneDB requirements were considered true microRNAs. MicroRNA annotations for *Exaiptasia pallida*, *Anemonia viridis*, *Acropora millepora*, *Acropora digitifera*, *Stylophora pistillata* and *Catalaphyllia jardinei*, *Nematostella vectensis*, and *Hydra magnipapillata* were obtained from previous studies [12, 36, 39] (Additional File 1: Table S16). The mature arm and precursor sequences of all cnidarian microRNAs were used as BLASTn queries against genomes, while mature miRNA sequences were also used as query sequences with MapMi to identify potential conserved miRNA loci in each cnidarian genome (MapMi scorer cutoff = 15)[86], followed by checking the hairpin structure with CentroidFold [87]. In this study, only conserved microRNAs found in at least three species were regarded as confident, and sequence alignments were further carried out with MUSCLE in MEGA7[88] and visualized in Jalview[81]. The identified cnidarian microRNAs were used as BLASTn queries against miRBase (v22) [41] (with *e*-value < 0.01) to confirm if there were any conserved microRNAs in bilaterians.

## Supplementary Information

Additional file 1: Tables S1–S16. Table S1-Genome and transcriptome sequencing data. Table S2-Information of Tripedalia maipoens genome assembly scaffolds. Table S3-Box jellyfish genomes statistics. Table S4-Genomes used for comparative genomic analyses. Table S5-KEGG pathway with gene gains identified in gene enrichment analyses. Table S6-Cnidopsin genes in Tripedalia maipoensis and Morbakka virulenta. Table S7-Jellyfish toxin genes in Tripedalia maipoensis and Morbakka virulenta. Table S8-Neuropeptides in different cnidarian genomes. Table S9-Sesquiterpenoid hormone biosynthetic pathway genes. Table S10-microRNAs in Tripedalia maipoensis. Table S11-microRNAs in Sanderia malayensis. Table S12-microRNAs in Chrysaora quinquecirrha. Table S13-microRNAs in Aurelia coerulea. Table S14-microRNAs in Rhopilema esculentum.Table S15-microRNAs in Mastigias papua. Table S16-Sample information for species used in microRNA analyses.

Additional file 2: Fig. S1-20. Fig. S1-Mean depth of Omni-C data in T. maipoensis genome. Fig. S2-Summary of T. maipoensis genome assembly v2 scaffolded by YaHS version 1.2.2. Fig. S3-Dot plots of genome alignments between v1 and v2 genome assemblies of T. maipoensis aligned by MashMap. Fig. S4-Summary of repeat annotations in the T. maipoensis genome. Fig. S5-Syntenic Oxford dot plots between T. maipoensis and 8 other taxa. Fig. S6-Pairwise comparison of macrosynteny mixing rate between 9 cnidarian genomes. Fig. S7-Synteny plot between T. maipoensis and M. virulenta. Fig. S8-Maximum-likelihood tree of UDP-glucuronosyltransferase (Pfam00201). Fig. S9-Maximum-likelihood tree of cnidopsins and other opsin genes. Fig. S10-Sequence alignment of cnidopsin group 1a. Fig. S11-Sequence alignment of cnidopsin group 1b. Fig. S12-Sequence alignment of cnidopsin group 2 and 3. Fig. S13-Summary of opsins in T. maipoensis and Morbakka virulenta. Fig. S14-Maximum-likelihood tree of jellyfish toxins (JFT). Fig. S15-Neuropeptides identified in T. maipoensis. Fig. S16-Sequence alignments of miR-100, miR-2022, miR-2030 and miR-2036. Fig. S17-Sequence alignments of conserved novel microRNAs in cubozoan, scyphozoans and anthozoans (miR-CC1 to miR-CC9). Fig. S18-Sequence alignments of conserved novel microRNAs in scyphozoans and cubozoan (miR-MC1 to miR-MC7). Fig. S19-Sequence alignments of conserved novel microRNAs in scyphozoans (miR-SC1 to miR-SC9). Fig. S20-Sequence alignments of miR-MC6 and miR-2023.

## Data Availability

The genome assembly of Tripedalia maipoensis is available at NCBI/ENA with accession JBDZFW000000000 [89], DDBJ with accession PRJDB40605, NGDC with accession GWHHPHM00000000, and CUHK Research Data Repository (https://doi.org/10.48668/QSFWRO). The raw reads generated in this study, including transcriptome, Omni-C and PacBio HiFi data, have been deposited and are publicly available at NCBI/ENA with accession PRJNA1117645, DDBJ with accession PRJDB40605, NGDC with accession PRJCA059704, and CUHK Research Data Repository (https://doi.org/10.48668/QSFWRO). The genome annotation files, code and scripts used in this study can be found at CUHK Research Data Repository (https://doi.org/10.48668/QSFWRO).

## Abbreviations

BUSCO: Benchmarking Universal Single-Copy Orthologs
JFT: Jellyfish toxin
LINE: Long interspersed nuclear element
TE: Transposable element
