# Supplementary material for: The first chromosome-level cubozoan genome differentiates unique and common features in different cnidarian lineages
Source: BMC Biol. 2026 Mar 24;24:107. doi: 10.1186/s12915-026-02579-7 (PMC13137736; doi:10.1186/s12915-026-02579-7)
Supplement: Supplementary file 2 — Additional file 2: Fig. S1-20. Fig. S1-Mean depth of Omni-C data in T. maipoensis genome. Fig. S2-Summary of T. maipoensis genome assembly v2 scaffolded by YaHS version 1.2.2. Fig. S3-Dot plots of genome alignments between v1 and v2 genome assemblies of T. maipoensis aligned by MashMap. Fig. S4-Summary of repeat annotations in the T. maipoensis genome. Fig. S5-Syntenic Oxford dot plots between T. maipoensis and 8 other taxa. Fig. S6-Pairwise comparison of macrosynteny mixing rate between 9 cnidarian genomes. Fig. S7-Synteny plot between T. maipoensis and M. virulenta. Fig. S8-Maximum-likelihood tree of UDP-glucuronosyltransferase (Pfam00201). Fig. S9-Maximum-likelihood tree of cnidopsins and other opsin genes. Fig. S10-Sequence alignment of cnidopsin group 1a. Fig. S11-Sequence alignment of cnidopsin group 1b. Fig. S12-Sequence alignment of cnidopsin group 2 and 3. Fig. S13-Summary of opsins in T. maipoensis and Morbakka virulenta. Fig. S14-Maximum-likelihood tree of jellyfish toxins (JFT). Fig. S15-Neuropeptides identified in T. maipoensis. Fig. S16-Sequence alignments of miR-100, miR-2022, miR-2030 and miR-2036. Fig. S17-Sequence alignments of conserved novel microRNAs in cubozoan, scyphozoans and anthozoans (miR-CC1 to miR-CC9). Fig. S18-Sequence alignments of conserved novel microRNAs in scyphozoans and cubozoan (miR-MC1 to miR-MC7). Fig. S19-Sequence alignments of conserved novel microRNAs in scyphozoans (miR-SC1 to miR-SC9). Fig. S20-Sequence alignments of miR-MC6 and miR-2023. [file 12915_2026_2579_MOESM2_ESM.docx]

**Additional File 2**

**Fig. S1.** Mean depth of Omni-C data in *T. maipoensis* genome.

**Fig. S2.** Summary of *T. maipoensis* genome assembly v2 scaffolded by YaHS version 1.2.2.

**Fig. S3.** Dot plots of genome alignments between v1 and v2 genome assemblies of *T. maipoensis* aligned by MashMap.

**Fig. S4.** Summary of repeat annotations in the *T. maipoensis* genome.

**Fig. S5.** Syntenic Oxford dot plots between *T. maipoensis* and 8 other taxa.

**Fig. S6.** Pairwise comparison of macrosynteny mixing rate between 9 cnidarian genomes.

**Fig. S7.** Synteny plot between *T. maipoensis* and *M. virulenta*.

**Fig. S8.** Maximum-likelihood tree of UDP-glucuronosyltransferase (Pfam00201).

**Fig. S9.** Maximum-likelihood tree of cnidopsins and other opsin genes.

**Fig. S10.** Sequence alignment of cnidopsin group 1a.

**Fig. S11.** Sequence alignment of cnidopsin group 1b.

**Fig. S12.** Sequence alignment of cnidopsin group 2 and 3.

**Fig. S13.** Summary of opsins in *T. maipoensis* and *Morbakka virulenta*.

**Fig. S14.** Maximum-likelihood tree of jellyfish toxins (JFT).

**Fig. S15.** Neuropeptides identified in *T. maipoensis*.

**Fig. S16.** Sequence alignments of miR-100, miR-2022, miR-2030 and miR-2036.

**Fig. S17.** Sequence alignments of conserved novel microRNAs in cubozoan, scyphozoans and anthozoans (miR*-CC1* to miR*-CC9*).

**Fig. S18.** Sequence alignments of conserved novel microRNAs in scyphozoans and cubozoan (miR*-MC1* to miR*-MC7*).

**Fig. S19.** Sequence alignments of conserved novel microRNAs in scyphozoans (miR*-SC1* to miR*-SC9*).

**Fig. S20.** Sequence alignments of miR-MC6 and miR-2023.

**
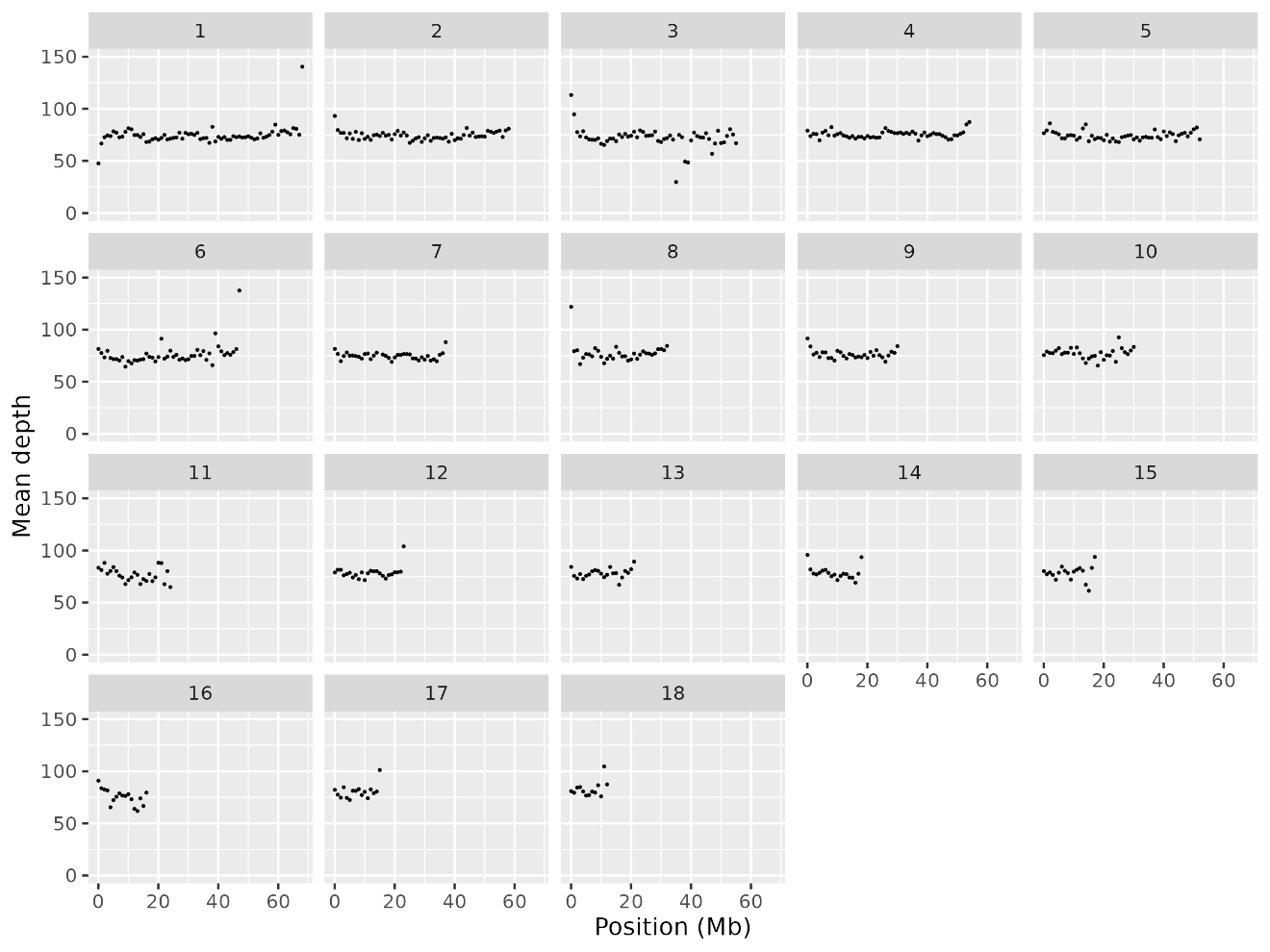
**

**Fig. S1.** Mean depth of Omni-C data in 1 Mb windows along the *T. maipoensis* 18 largest scaffolds.


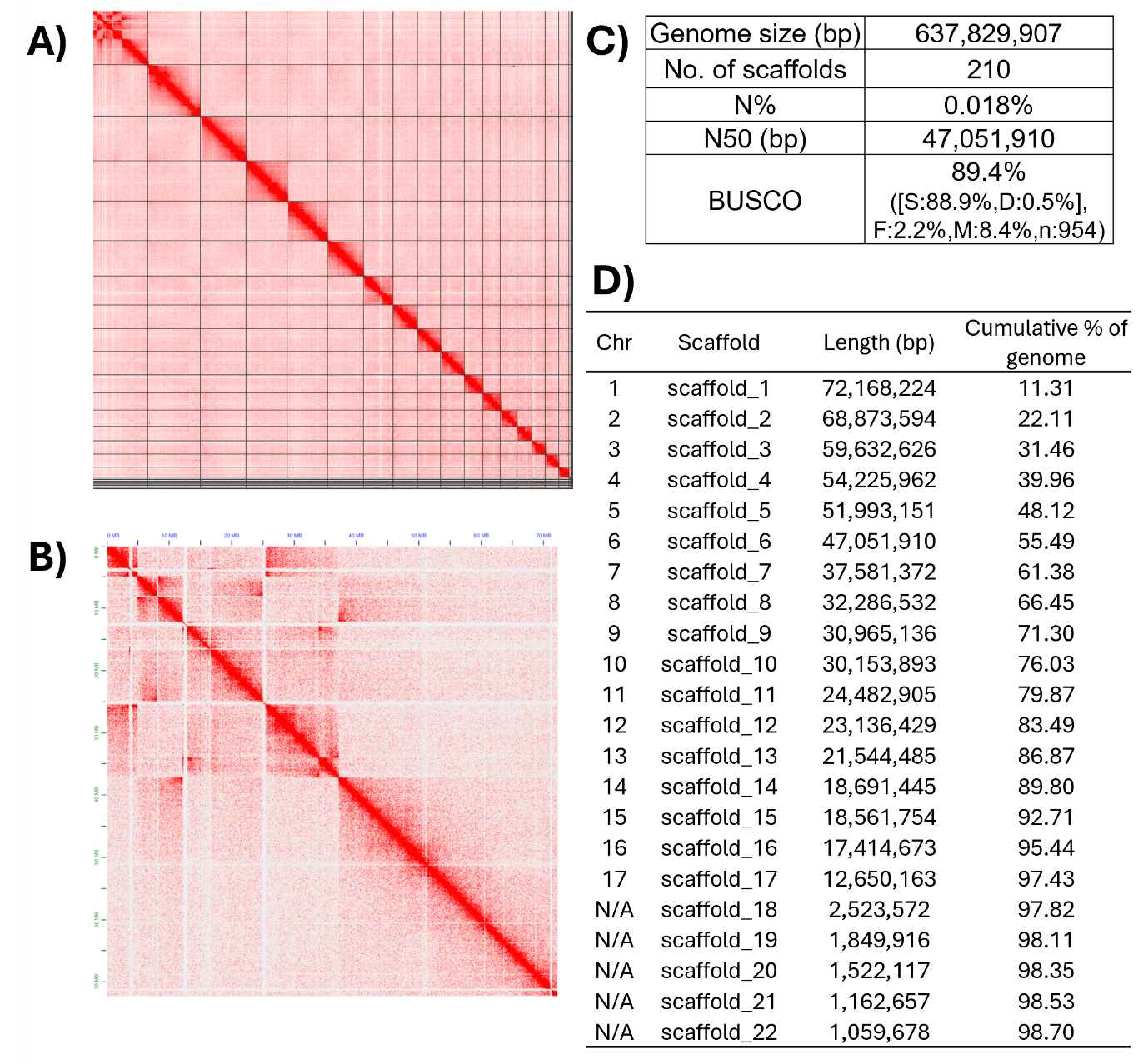


**Fig. S2.** Summary of *T. maipoensis* genome assembly v2 scaffolded by YaHS version 1.2.2. **A)** Omni-C contact map; **B)** Enlarged view of Omni-C contact map of chromosome 1; **C)** Genome statistics summary. Abbreviations for BUSCO statistics: D, duplicated complete BUSCO; F, fragmented BUSCO; M, missing BUSCO; n, total number of BUSCO in dataset metazoa_odb10; S, single-copy BUSCO; **D)** Scaffold information of *T. maipoensis genome* assembly v2. Scaffolds with lengths larger than 1 Mb are shown.


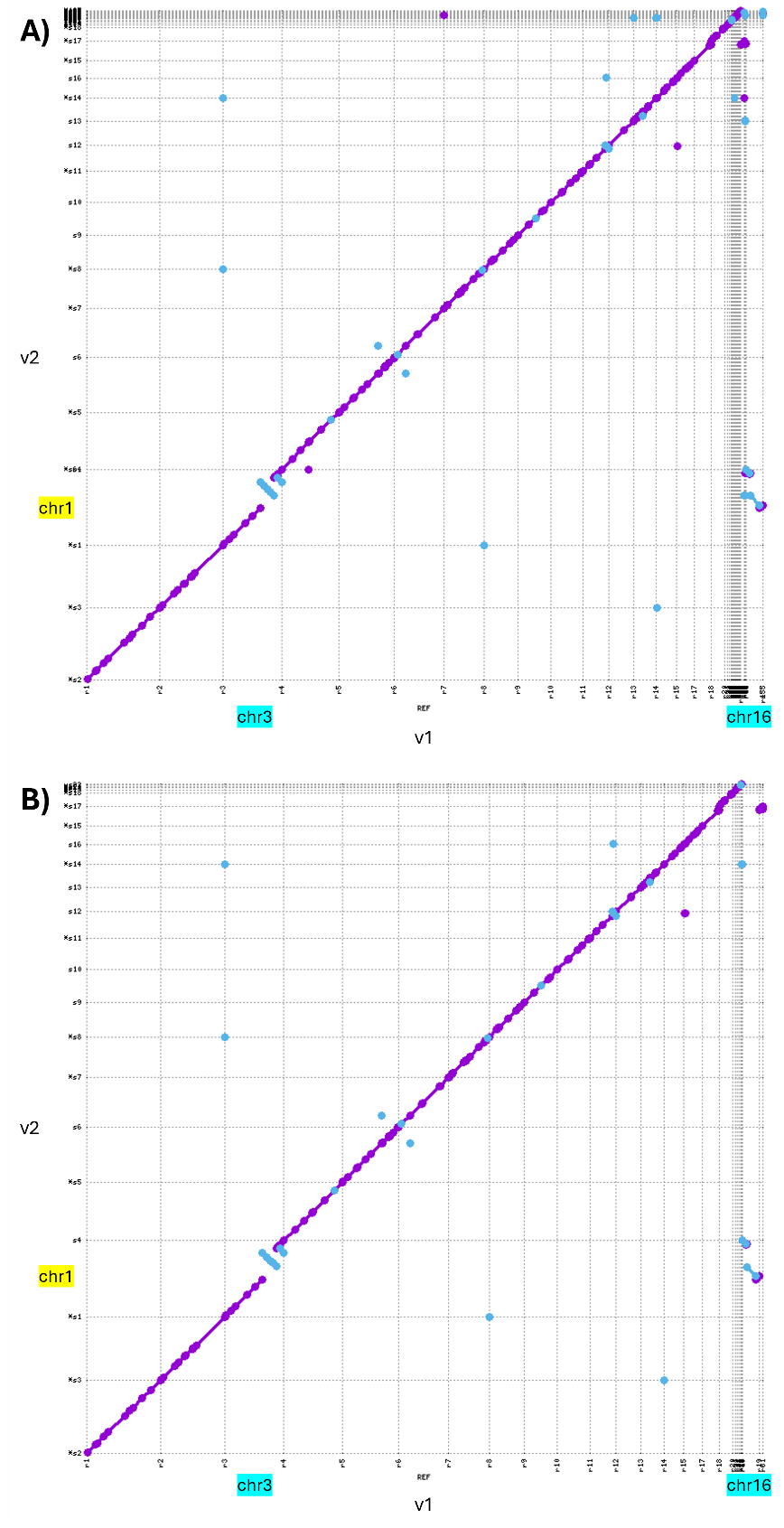


**Fig. S3.** Dot plots of genome alignments between v1 and v2 genome assemblies of *T. maipoensis* aligned by MashMap, where all scaffolds (**A)** and scaffolds with length larger than 100 kb **(B)** were used. Chromosomes 3 and 16 of v1 genome assembly (x-axis) were highlighted in blue while chromosone 1 of v2 genome assembly (y-axis) was highlighted in yellow.

**
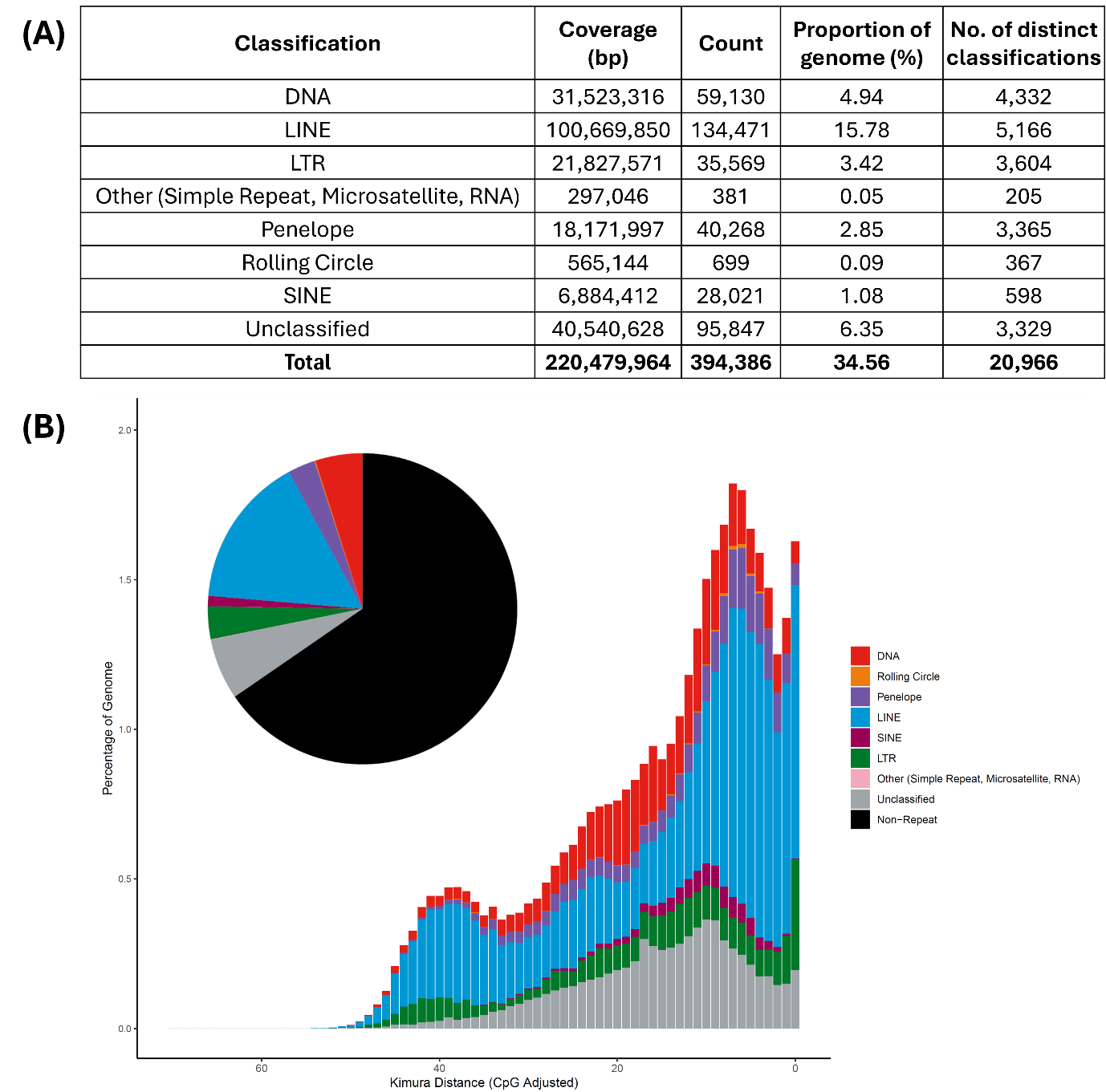
**

**Fig. S4.** Summary of repeat annotations in the *T. maipoensis* genome.

**
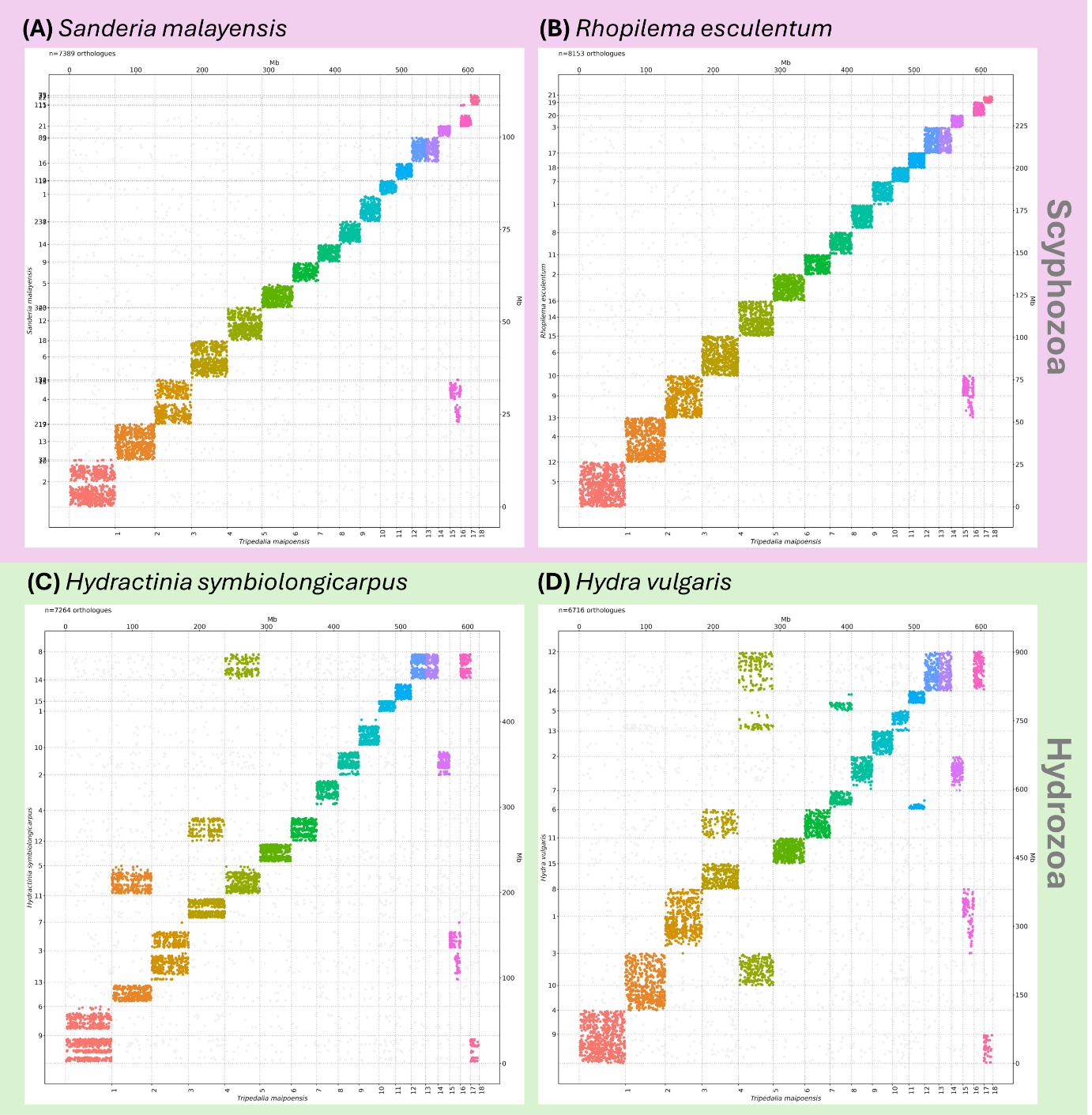

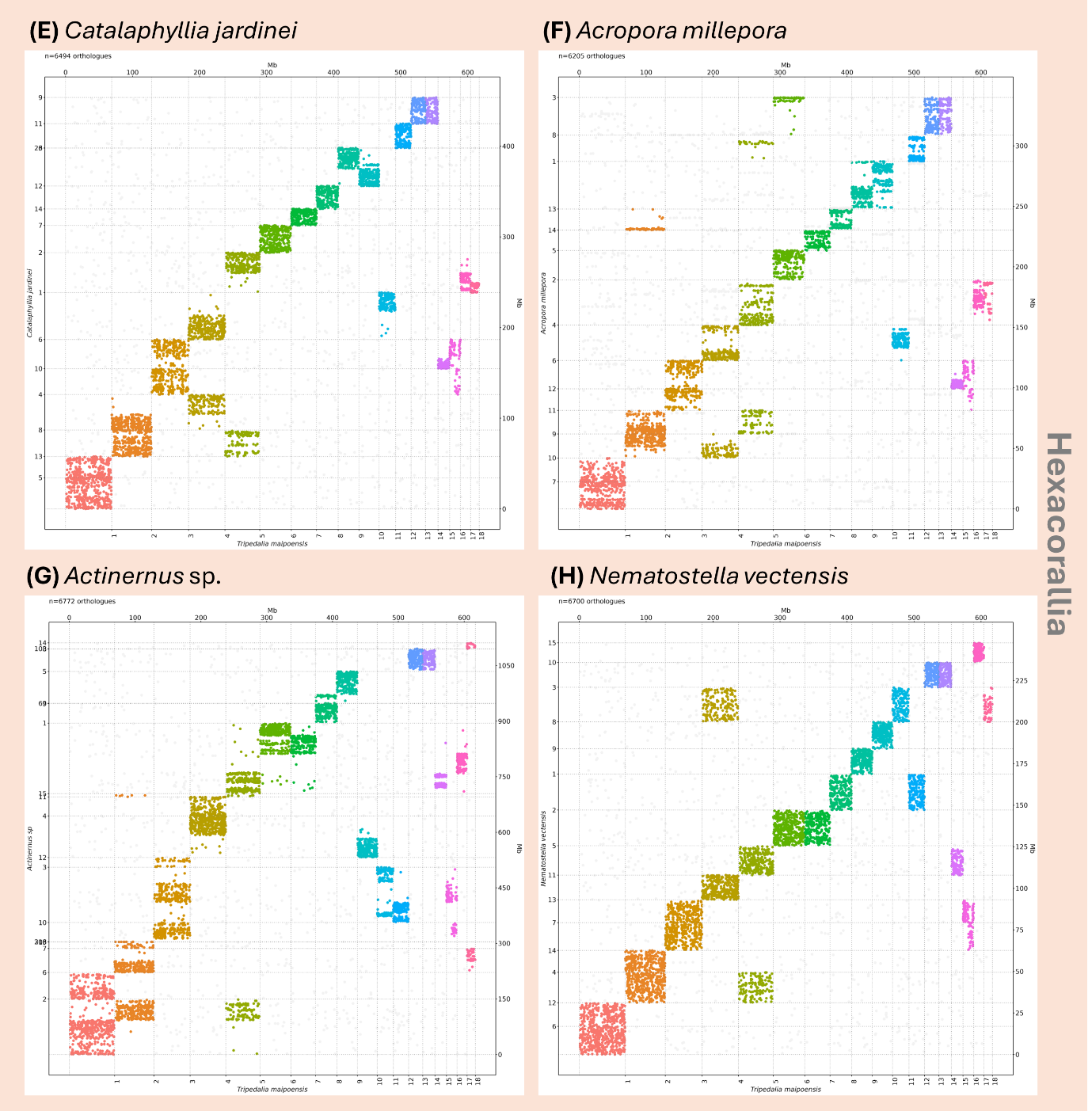
**

**Fig. S5.** Syntenic Oxford dot plots between *T. maipoensis* (x-axis) and 8 other taxa (y-axis), namely **A)** *Sanderia malayensis*, **B)** *Rhopilema esculentum*, **C)** *Hydractinia symbiolongicarpus*, **D)** *Hydra vulgaris*, **E)** *Catalaphyllia jardinei*, **F)** *Acropora millepora*, **G)** *Actinernus* sp., and **H)** *Nematostella vectensis*.


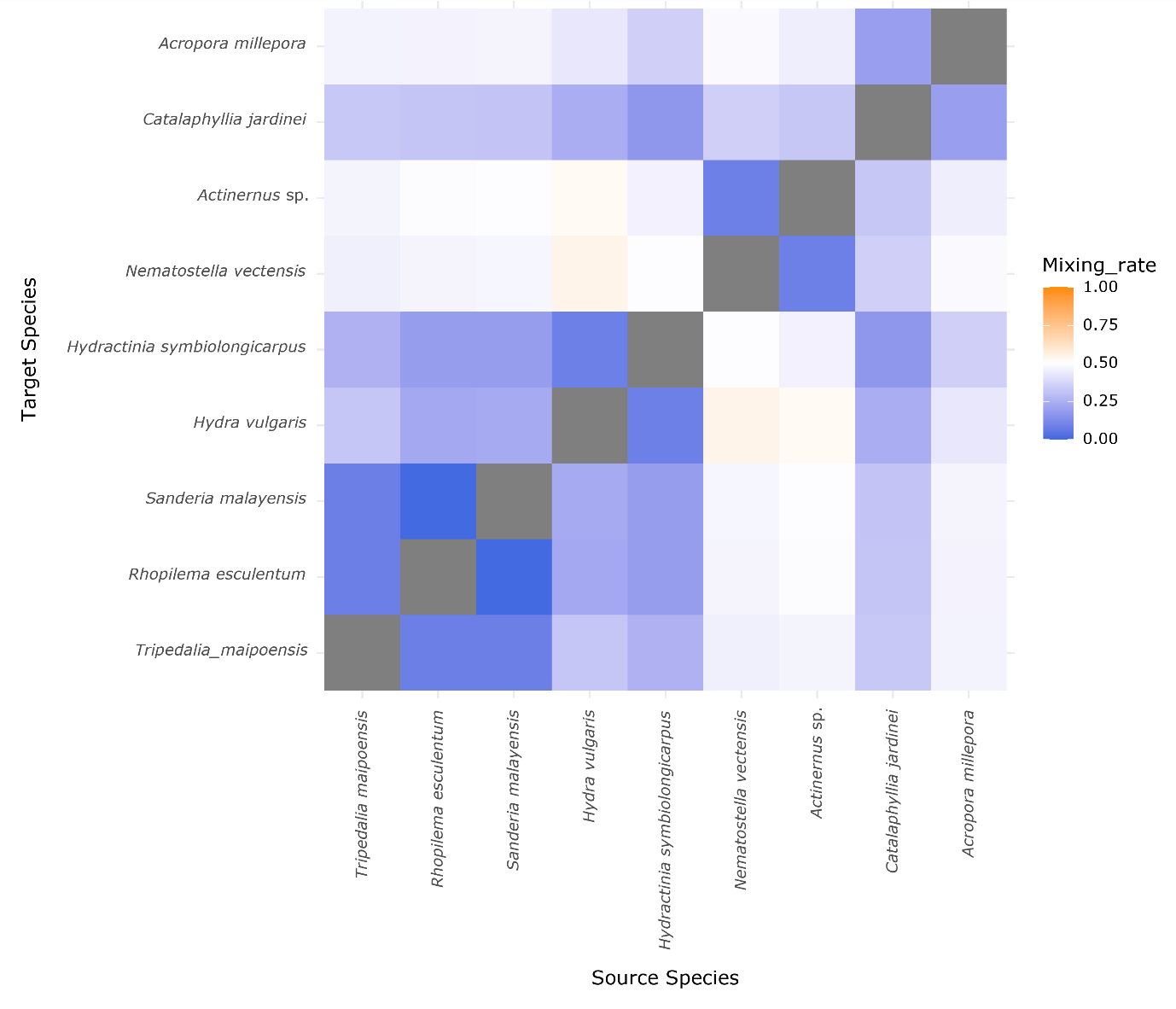


**Fig. S6.** Pairwise comparison of macrosynteny mixing rates between 9 cnidarian genomes.

**Fig. S7.** Synteny plot between *T. maipoensis* (Tmai) and *M. virulenta* (Mvir).

**Fig. S8.** Maximum-likelihood tree of UDP-glucuronosyltransferase (Pfam00201) constructed from domain sequences with 1,000 bootstraps.


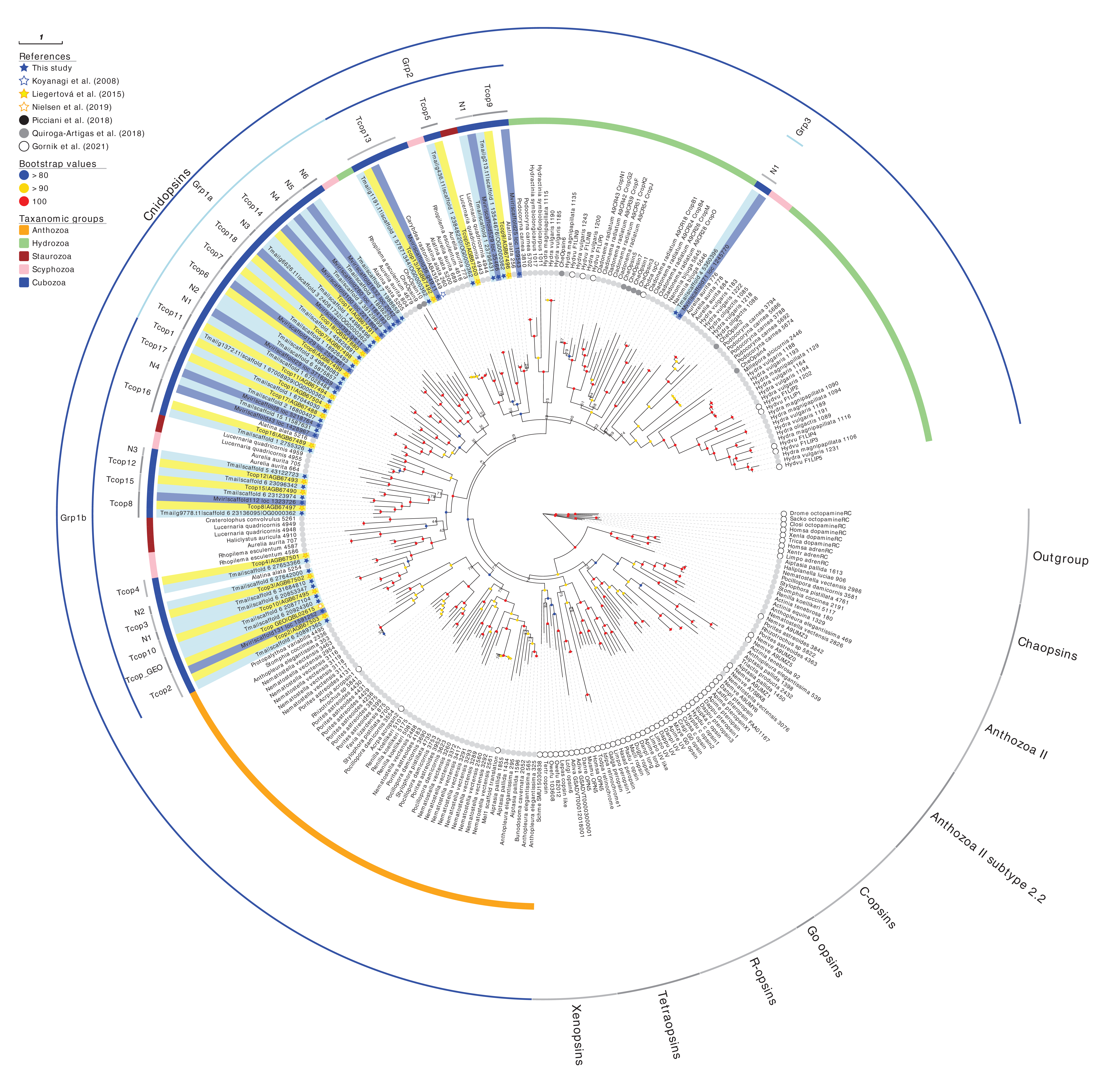


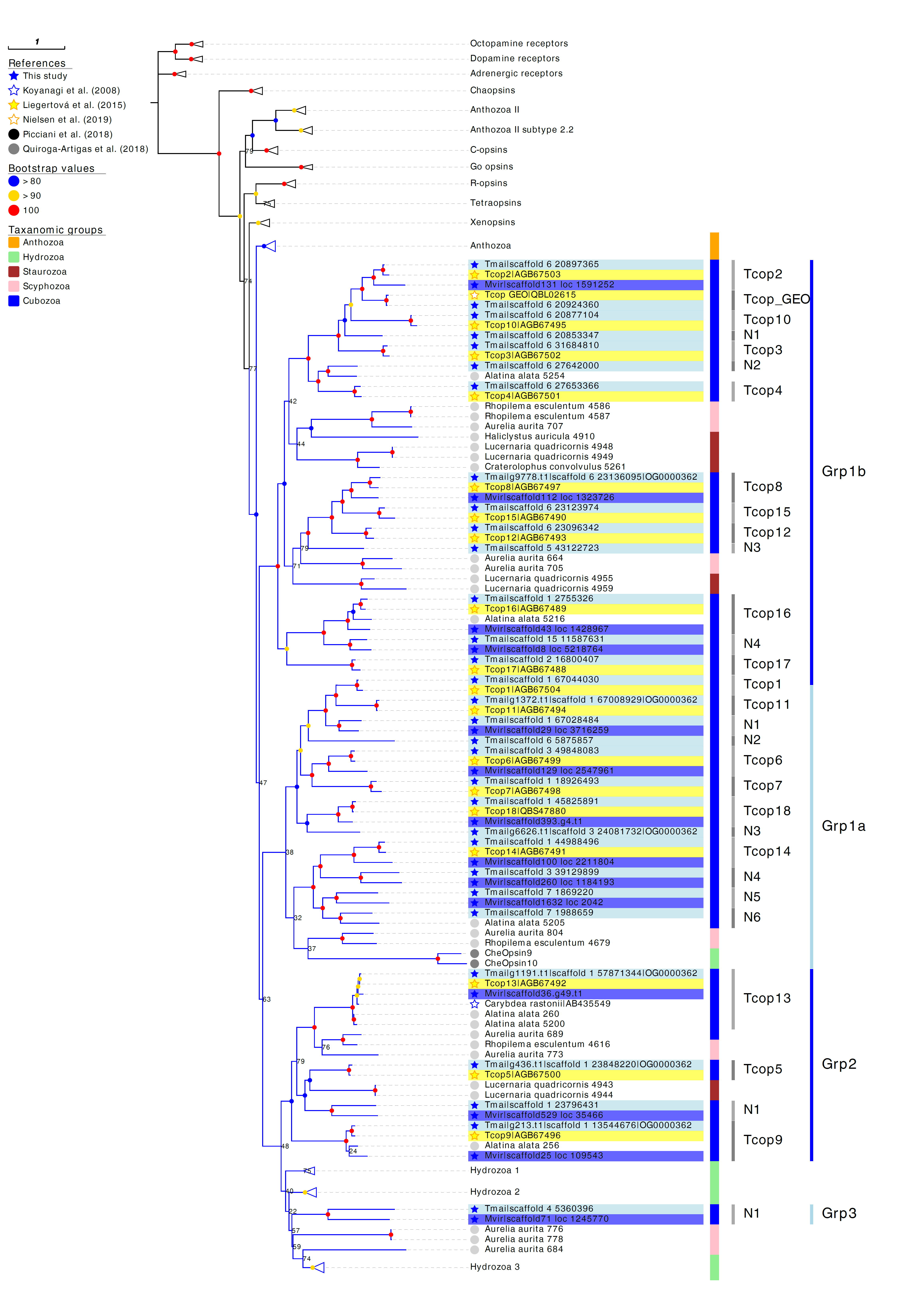

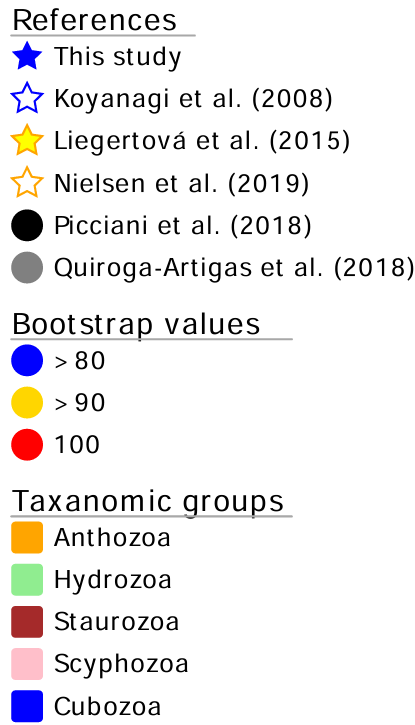


**Fig. S9.** Maximum-likelihood tree of cnidopsins and other opsin genes constructed with 1,000 bootstraps visualized in circular (upper panel) and rectangular (lower panel) forms. Cnidopsins identified from *Tripedalia maipoensis* and *Morbakka virulenta* are highlighted in lightblue and blue, respectively, while the reference sequences from *Tripedalia cystophora* are highlighted in yellow. The outermost annotated ring describes the grouping of cnidopsins and other G protein-coupled receptors, followed by an inner and innermost annotated ring of the grouping and gene orthology of *Tripedalia cystophora* cnidopsins as described in Liegertová et al. (2015). For outgroup, octopamine receptors, dopamine receptors and adrenergic receptors were used as described in Gornik et al. (2021). The outgroups and opsins other than cnidopsins are concatenated in the rectangular tree.


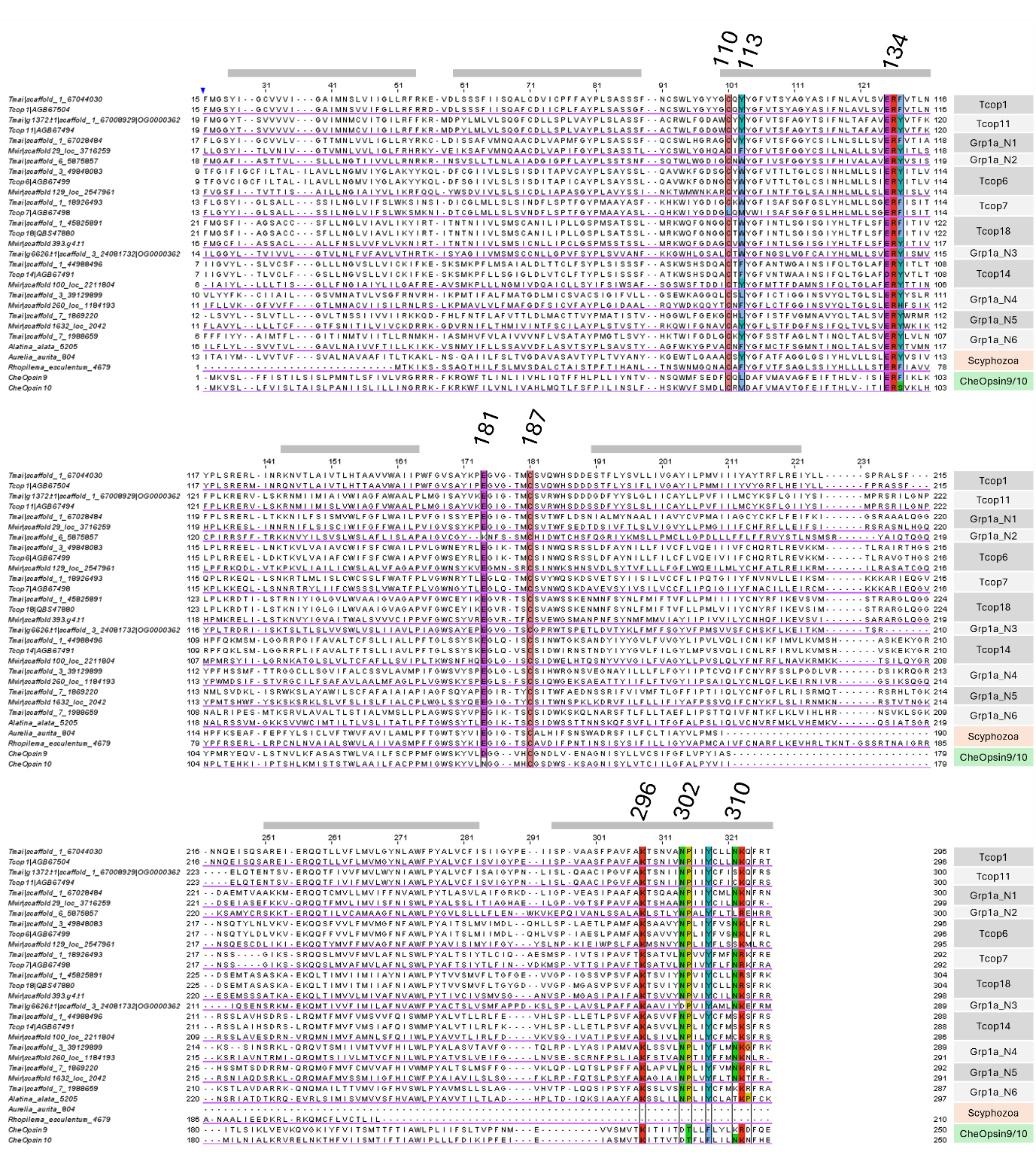


**Fig. S10.** Sequence alignment of cnidopsin group 1a. The sequences are sorted according to the phylogenetic tree topology of the group 1a. The seven conserved transmembrane domains are indicated by grey rectangles and the trimmed regions of both ends are shown with blue triangles above the sequence alignment. The numbers on top of the sequence alignment denote the conserved structural and functional opsin motifs corresponding to the bovine rhodopsin numbering system as adopted in Gornik et al. (2020), which include (1) two cystine [C] residues at positions 110 and 187 for the formation of disulphide bond; (2) two residues at positions 113 and 181 acting as negative counterions that stabilise the proton on the Schiff base; (3) a negative charge residue at position 134 to stabilise the inactive opsin molecule, as part of the conserved motif of “E/DRY” (positions 134-136) in rhodopsin; (4) a conserved lysine [K] residue at position 296 for a covalent linkage with the 11-cis retinal chromophore via a Schiff base; (5) a conserved NPxxY motif from position 302 to 306 and an NKQ motif (310–312) in rhodopsin that involve in maintaining the structural integrity upon photopigment activation. Cubozoan cnidopsins that are orthologous to the 18 cnidopsins in *Tripedalia cystophora* (Tcop1-18) described by Liegertová et al. (2015) are labelled on the right panel.


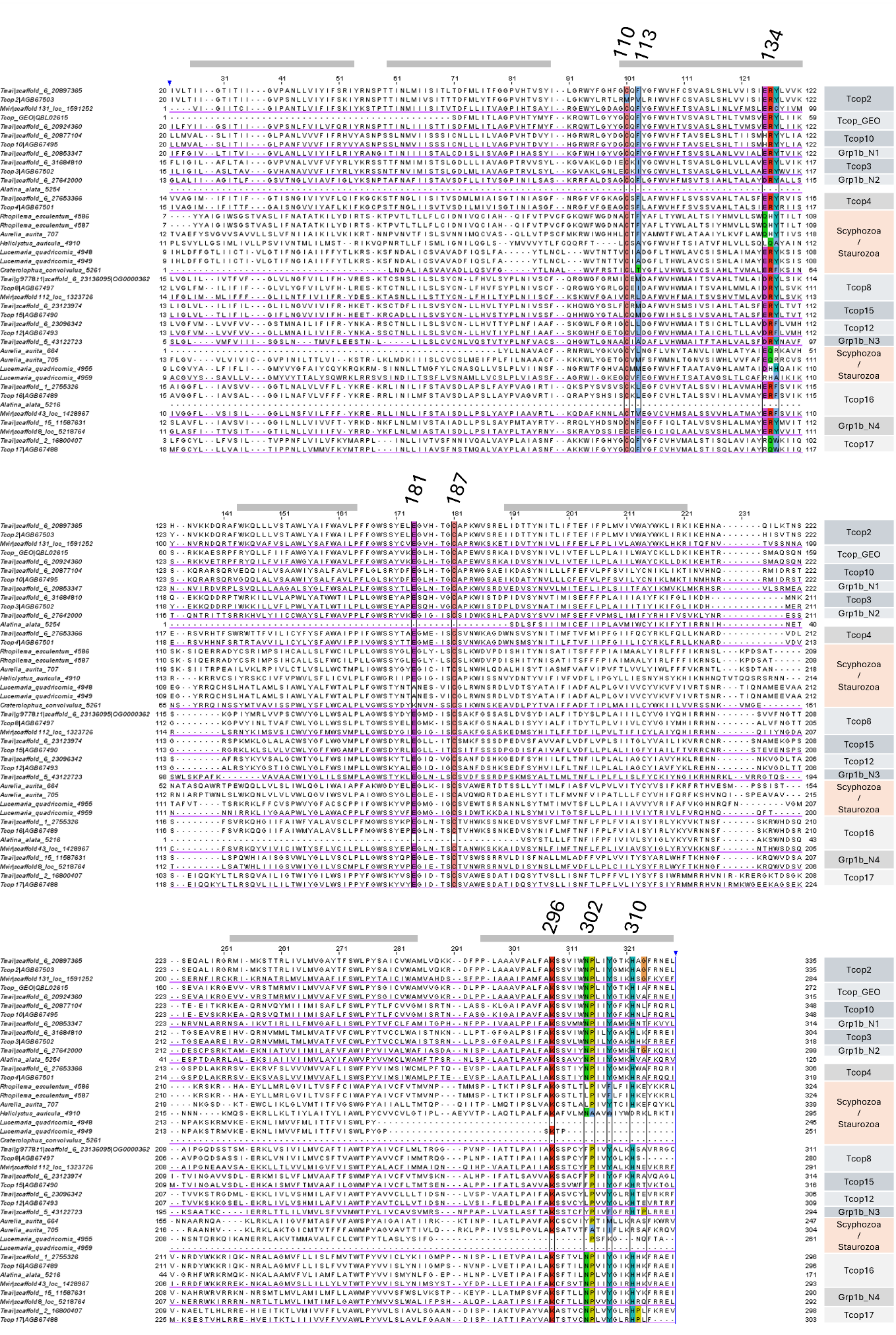


**Fig. S11.** Sequence alignment of cnidopsin group 1b. The sequences are sorted according to the phylogenetic tree topology of the group 1b. The seven conserved transmembrane domains are indicated by grey rectangles and the trimmed regions of both ends are shown with blue triangles above the sequence alignment. The numbers on top of the sequence alignment denote the conserved structural and functional opsin motifs corresponding to the bovine rhodopsin numbering system as adopted in Gornik et al. (2020), which include (1) two cystine [C] residues at positions 110 and 187 for the formation of disulphide bond; (2) two residues at positions 113 and 181 acting as negative counterions that stabilise the proton on the Schiff base; (3) a negative charge residue at position 134 to stabilise the inactive opsin molecule, as part of the conserved motif of “E/DRY” (positions 134-136) in rhodopsin; (4) a conserved lysine [K] residue at position 296 for a covalent linkage with the 11-cis retinal chromophore via a Schiff base; (5) a conserved NPxxY motif from position 302 to 306 and an NKQ motif (310–312) in rhodopsin that involve in maintaining the structural integrity upon photopigment activation. Cubozoan cnidopsins that are orthologous to the 18 cnidopsins in *Tripedalia cystophora* (Tcop1-18) described by Liegertová et al. (2015) are labelled on the right panel.

**
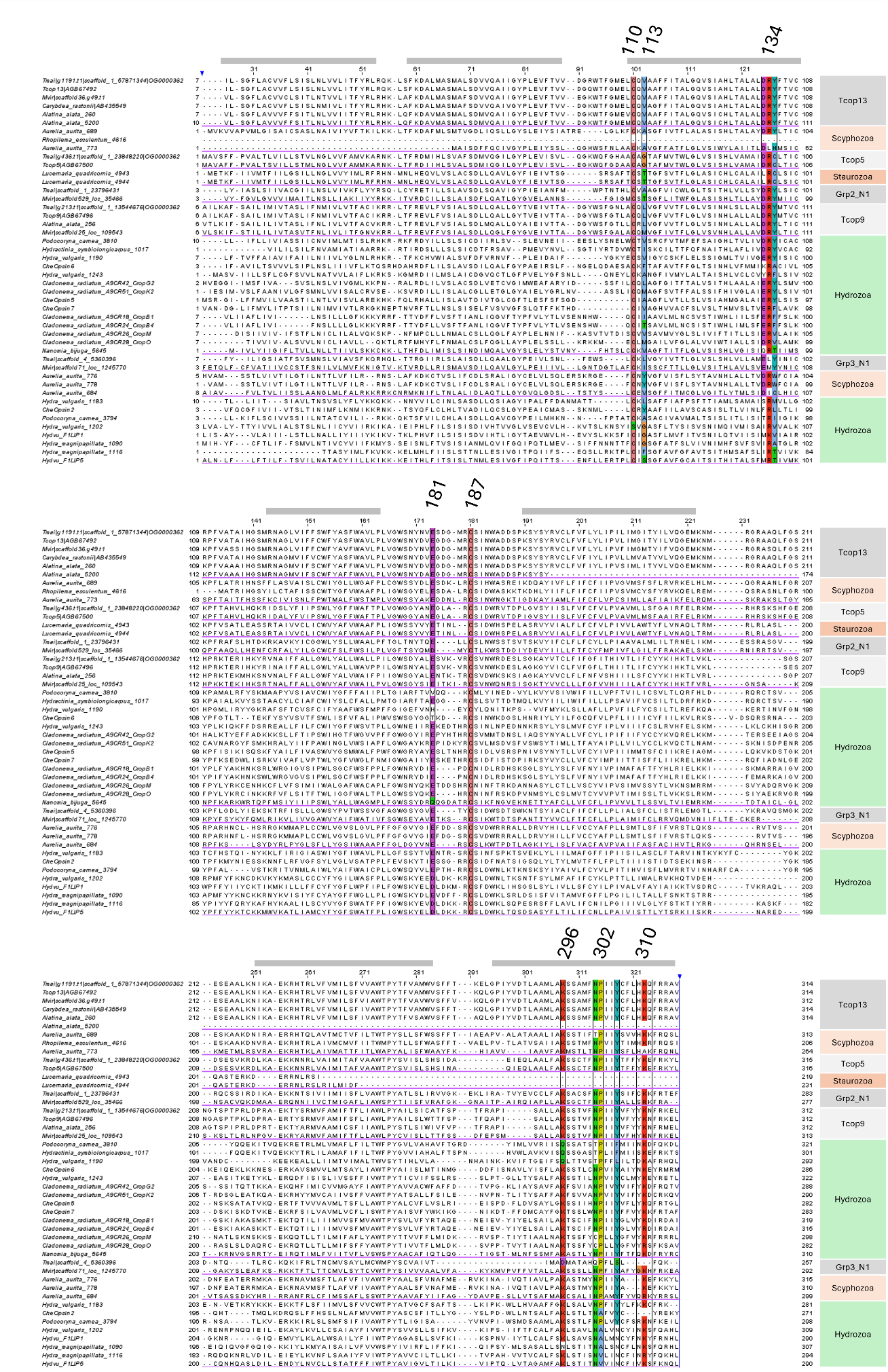
Fig. S12.** Sequence alignment of cnidopsin group 2 and 3. The sequences are sorted according to the phylogenetic tree topology of the group 2 and 3. The seven conserved transmembrane domains are indicated by grey rectangles and the trimmed regions of both ends are shown with blue triangles above the sequence alignment. The numbers on top of the sequence alignment denote the conserved structural and functional opsin motifs corresponding to the bovine rhodopsin numbering system as adopted in Gornik et al. (2020), which include (1) two cystine [C] residues at positions 110 and 187 for the formation of disulphide bond; (2) two residues at positions 113 and 181 acting as negative counterions that stabilise the proton on the Schiff base; (3) a negative charge residue at position 134 to stabilise the inactive opsin molecule, as part of the conserved motif of “E/DRY” (positions 134-136) in rhodopsin; (4) a conserved lysine [K] residue at position 296 for a covalent linkage with the 11-cis retinal chromophore via a Schiff base; (5) a conserved NPxxY motif from position 302 to 306 and an NKQ motif (310–312) in rhodopsin that involve in maintaining the structural integrity upon photopigment activation. Cubozoan cnidopsins that are orthologous to the 18 cnidopsins in *Tripedalia cystophora* (Tcop1-18) described by Liegertová et al. (2015) are labelled on the right panel.


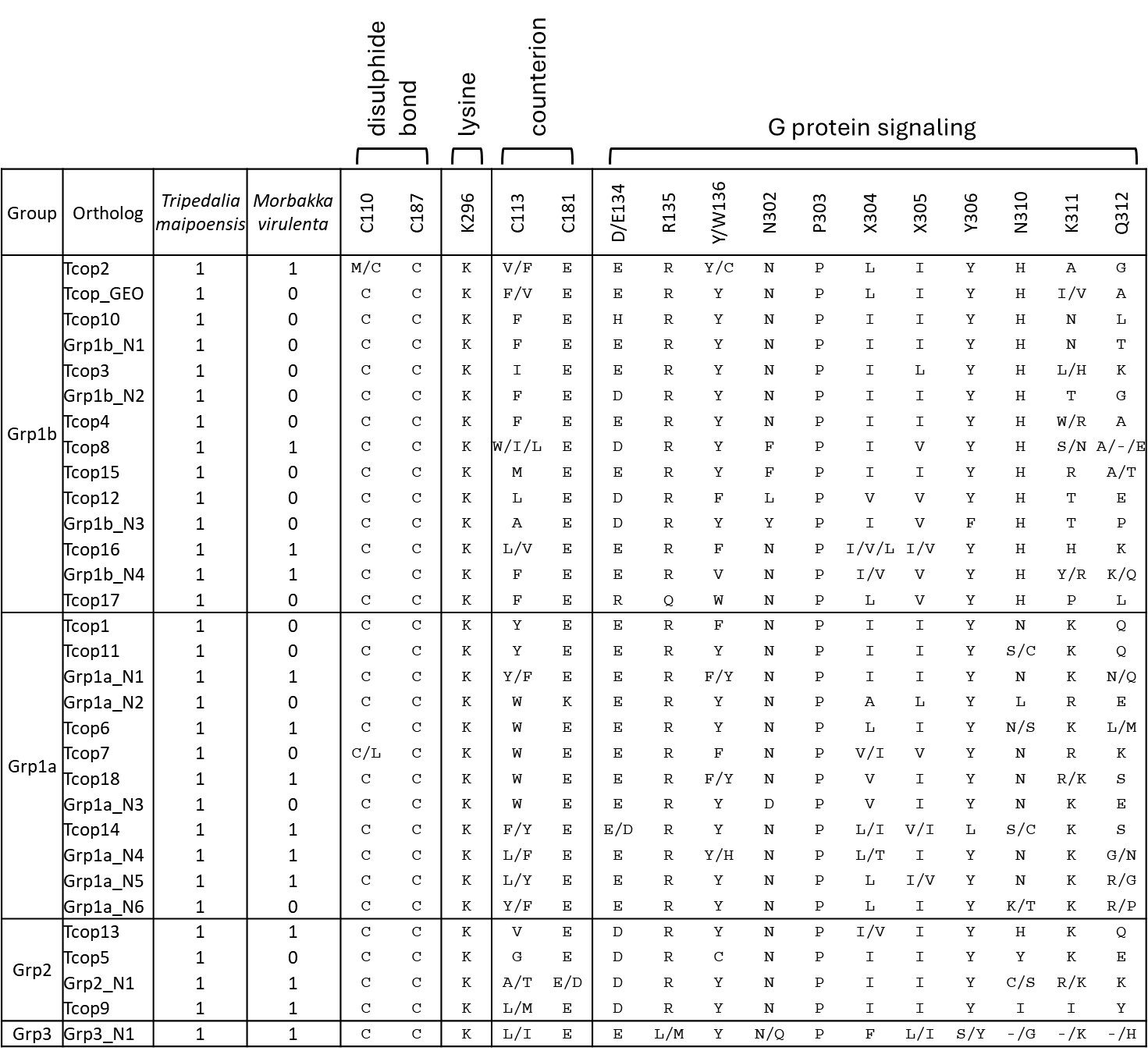


**Fig. S13.** Summary of the conserved structural and functional opsin motifs in the 31 cubozoan cnidopsin orthologues identified from *T. maipoensis* and *Morbakka virulenta*. The conserved structural and functional opsin motifs are summarized from Supplementary Figures 8-10.


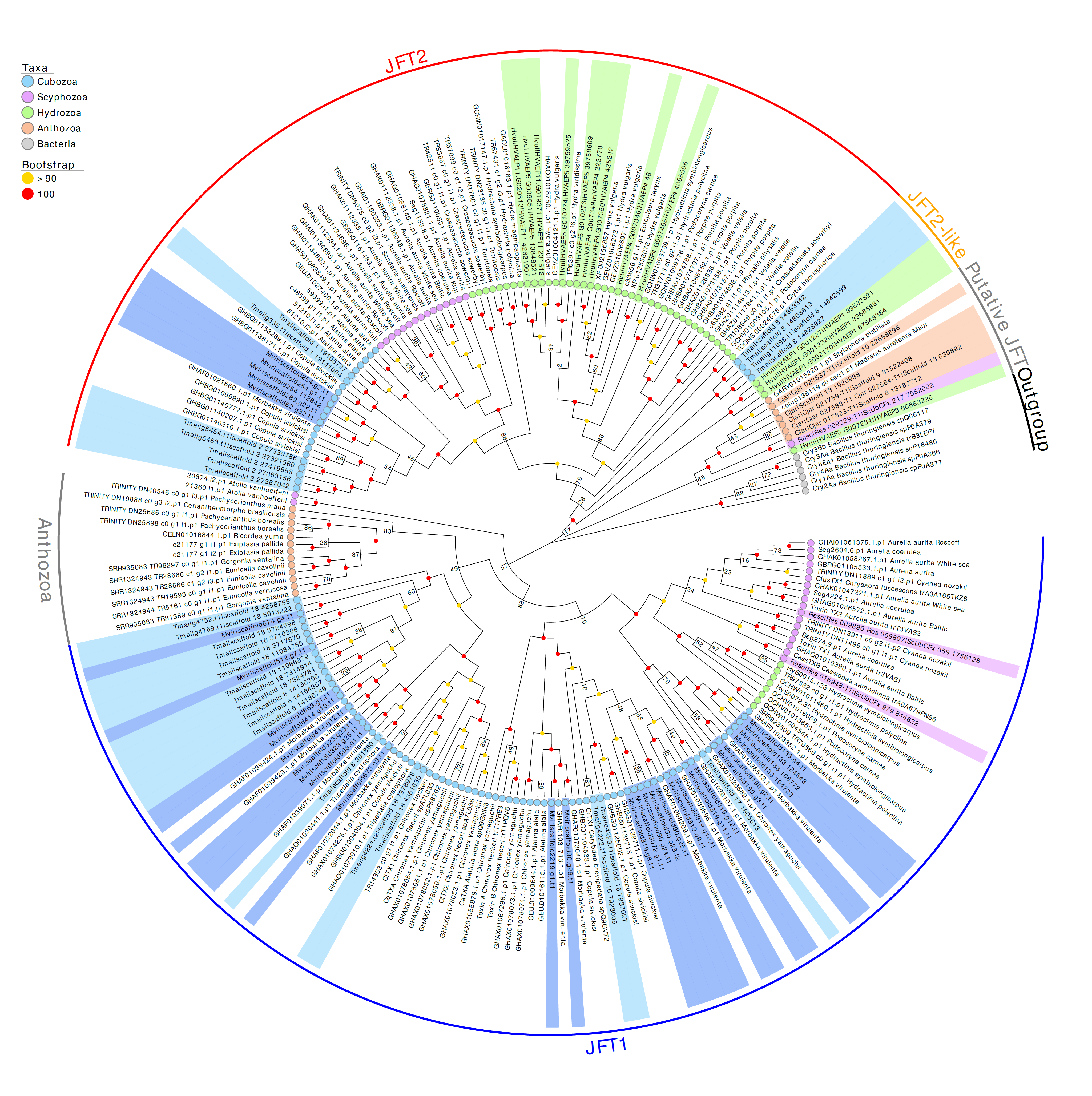

**Fig. S14.** Maximum-likelihood tree of jellyfish toxins. Jellyfish toxins (JFTs) identified from *Tripedalia maipoensis*, *Morbakka virulenta*, *Rhopilema esculentum*, *Hydra vulgaris* and *Catalaphyllia jardinei* in this study are highlighted in lightblue, blue, purple, green and orange, respectively. The reference sequences of cnidarian JFTs and outgroup (bacterial Cry toxins) were extracted from Klompen et al. (2021).

Predicted signal sequence and cleavage sites

>g9373.t1_FRamide

MNKPSMVLQRALWFTFFAVTLTCKVATADAEESTKSETYENWTTDEEIENQVPVSQNTPCQGEVCLYGNRQLARRKLLKRKTVCGQGGCLLNPEAKSIDHELDAAEMDTYGIQYNQLPRDSSCQGQMCWFRGKREKFTASNIQERLLAQLSFLRELNKKEMNVNNGKRAQGELGCTGQMCWFRGKKEYDESQDQSSCVGQMCWFRGKRTLNTKGSKSCTGQMCWFRGKRNMLTKDGALKKELNEQKRCTGQMCWFRGGRSLDEKGVDGKAARQKGKRSKKEDMCTGQMCWFRGGRSLDEKGVNGVAANQNGKRTQKETMCTGQMCWFRGRKELQAHQMGCKGQMCWFRGKRNAKHEIKN

>g5906.t1_VWamide

MMKAPSYKIGLVVLMLQLIVSLHVTCATDMNEESATSPVKRARSEEEVQEDAVVKALERGIFARRDVISQPPGVWGRSSERSFRARAFNRDRHLFRPQSPAERRALQDSIEHLLQAKEALDVLNKRASQPPGVWGKRVAAQPPGVWGKRTVRTQPPGVWGKRTVRTQPPGVWGKRALEFMEREYALGQVESKDEDTIAARSASVSQPPGVWGRRSIERSHSEEQ

>g1334.t1_RFamide

MELNTKALVLAFLAIAIVCATAADTKLAQQSRRQEEEDSQLFLSEEDDLVDERSVNKAKRIIEQWLRGRFGKRDLRGRFGREADSGDTVTENKARETLSQWLRGRFGREASDQWLRGRFGREATAQWLRGRFGREAMDQWLRGRFGREVADQWLRGRFGREVNSQWLRGRFGREVATQWLNGRFGREVADQWLRGRFGRQVEEDIEDELFDRELDQWLRGRFGRGTEQWLNGRFGKRESNEQWLRGRFGREMDQWLRGRFGREAEQWLRGRFGREMEQWLRGRFGREVEQWLRGRFGREAEQWLRGRFGRESNESEDKVEQWLRGRFGRNLEQGHLGVENSQGNGMEPNVARAVKTEDRSATVGVKDQRQDSSQVEAELSGQGSISDKRNL

>g9177.t1_LWamide

MWASYYAAPFLGLIFMLAIASAAPREDTEDQSASKRDASDKEQMGYKANTVRETLIKGYPNRPSVLWGRDVVAKDNLKNTEISEGLQPGMWGKRSLSQPRLNMLWGRAVVENPNMGLWGREKEMFERPKVGLWGRSSSPGKLGLWGRQLQESGYQPRDRPLGGLWGKEIRARDVENNQGSSDELERELENKIISLFSQLKSAKERREATEKRKPGVVGIWGK

>g9270.t1_RAamide

MSGIYFFLLLGIGTYFLCQSFAKGECNTDSTEYAYKRSDCLEDLLGLEPTEFADFLEEQPRSGKRELSVQDYRPRAGREGDQDYRPRAGRQTLTRPRGGREYSARPRAGREYTIKIISSEMQGFDRPRAGRESAARPRAGRENIQRPRAGRESLNRPRAGRENLERPRAGRENLERPRAGREYEGRPRAGREYEGRPRAGREYEGRPRAGREYEGRPRAGRQMLGRPRAGREFLERPRAGRNVVLVLNDGDNEGSNKRSFYIRSKVHAESQQDVSRTQEEEKGGELLDERESDHVVDAMDEEAANFMNENQPRSGKRELSGKAALALRLKREGEAIRSAMGKISGSTFAASSALEDEMNNQPRSGKRSILLGKHGIGSLQAGKKDSTDMKRGLNFPVEEDGQKLVFLMANKRENKKNDISKELEKLAGSLSKRRREVV

>g10227.t1_RY-precursor

MAKRLIFSAFLAIFICLVEAKAWKRESNHHFAKGSKDPQWPRYKKSESNHDPQWPRYRKSELEHKDQWPRYKKSQERNSPQWPRYKKSEALQWPRYKKSEIFASEQWPRYKKSEVKHSPQWPRYKKSELRKSPQWPRYKKEASKSAHWPRYKKSEAIRPAQWPRYKKSEASKSAHWPRYKKSEASRPAQWPRYKKSEAESEAQWPRYRK

>g1341.t1_RYamide

MVKIYIFALFCVAYVQSAVISTYIVRDVNPRDLHNHDMIFTDDSSSQGVKKREATLMKANTNTWNRMAFKHQRERRDTPPWVKGRYGRGVFHQNLRQLREGAVKVPREDYEKKVMAFLLNNRLAREADKFHGQLYARQMWHHQRYGREGKNGRNQGAPISPFVDEEKRITDEEKNILKGSFDKRERAAPGWHHGRYGRRVYGYYQGSMKRETPVWAKGRYGKREKEQDKHDLTQRYRKEDDEQDVEEIYDEGEMEHSEDGLAENSQLYRLSQRANH

>g3266.t1_RRFamide

MSAIIKLYQSQDLFQSIRCLHLKMNQQTQAFLLLVTLCIVFQNGCSLENSQLEEVSYRVSTDANADFLLCRDMFITSAVKRLAHSRMRDEWTVENSDYQKKRRGRFGKRTLGNGNWKQESPQHFHDNSGKLEKIARQIIAFCQFKILDSDERELDAFKRDRHDFRHDEAEEKKSSLSQSSRKSSEKFLEGDDPIWLPLATAMMTENDRELGAYNIQESKEEDDAYDMIDQHLAAFDSMAKKETAGMKPNRRRFGKKEVISAGSNTKPTRRRFGKEVSRGSTNLSQRSTNEEKSREAVIGETSLPQEKPRKRISKRASKTDYQRRRFGKREKNESYRMFTNVPHNRRFGKREDSENYLMVADLPQNRRFGKREDGDEYLGNEYYGKILGDNRKEQD

Annotations: Signal sequence Cleavage site

**Fig. S15.** Neuropeptides identified in *T. maipoensis*.

**miR-100:**

**
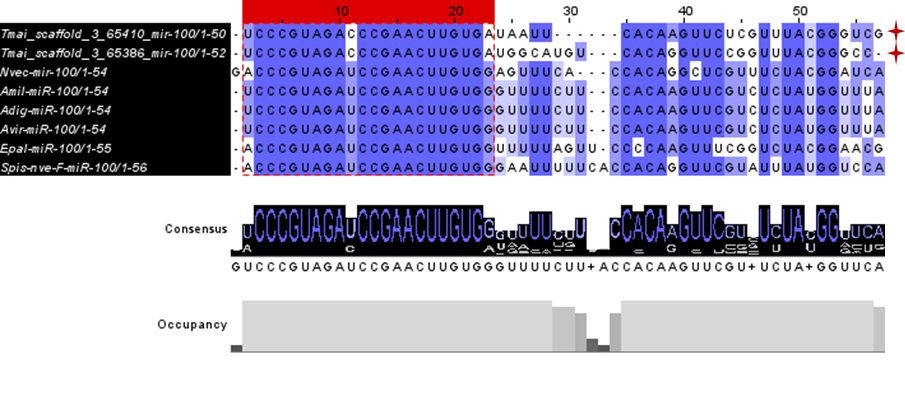
**

**miR-2022:**

**
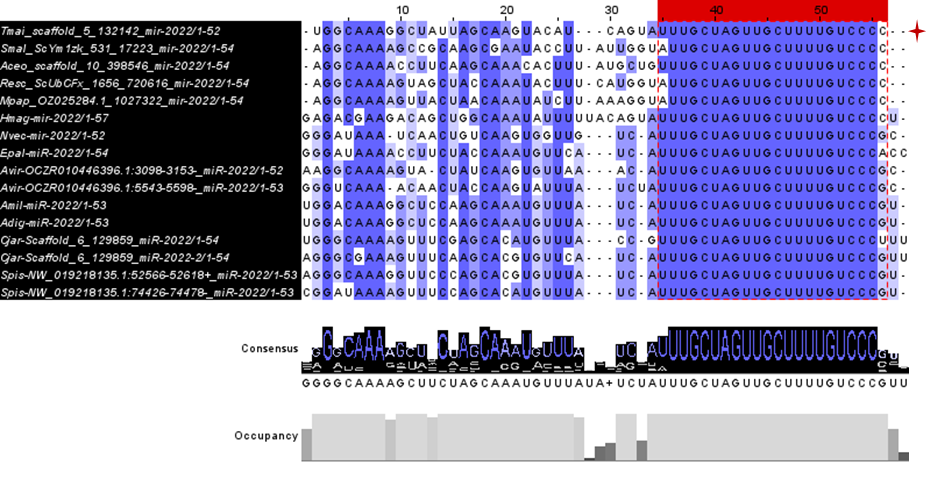
**

**miR-2030:**

**
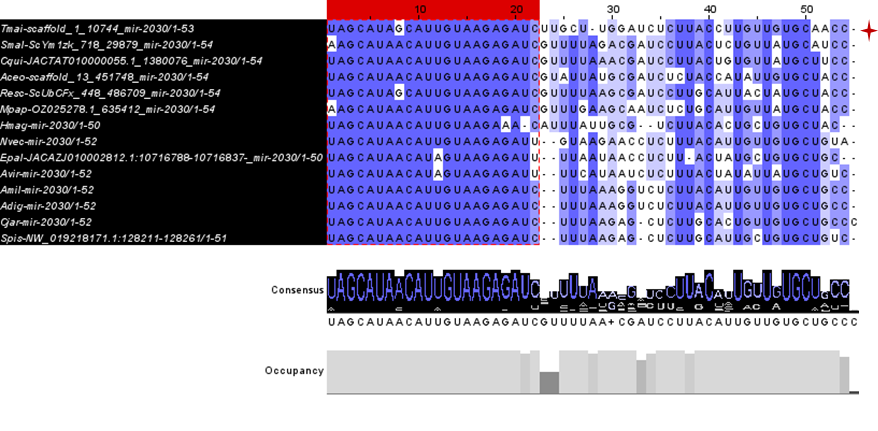
**

**miR-2036:**

**
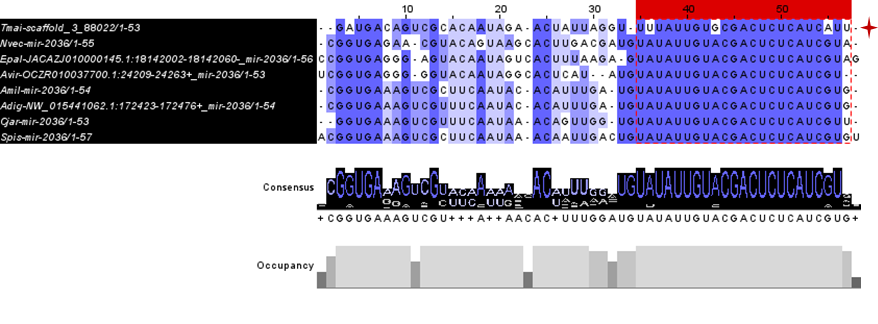
**

**Fig. S16.** Sequence alignment of miR-100, miR-2022, miR-2030 and miR-2036.

miR*-CC1*:


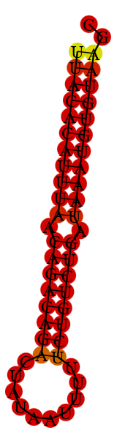
Ref: *Smal*_ScYm1zk_906_45824


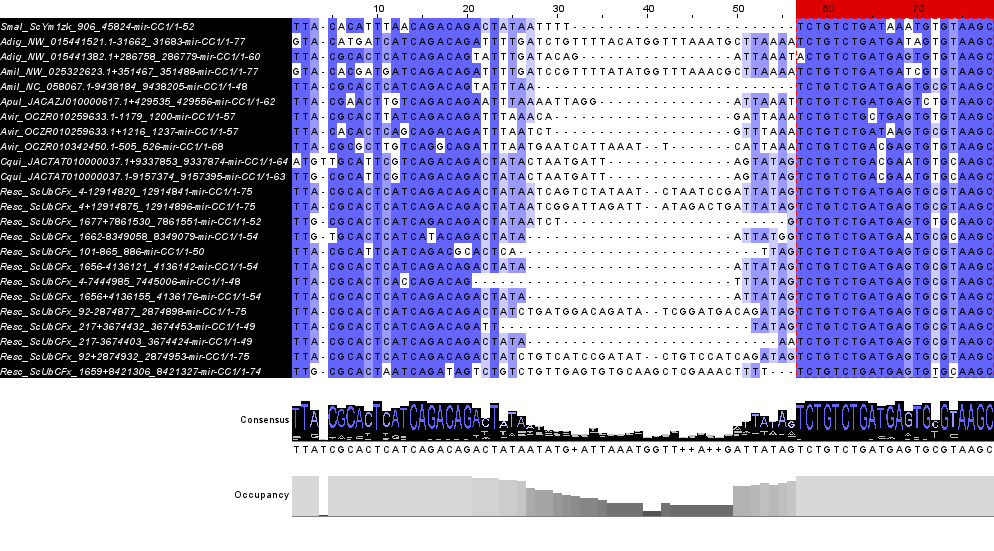


miR*-CC2*:


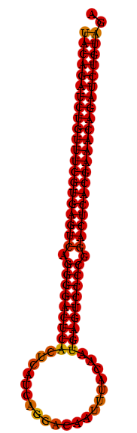
Ref: *Tmai*_scaffold_2_60676


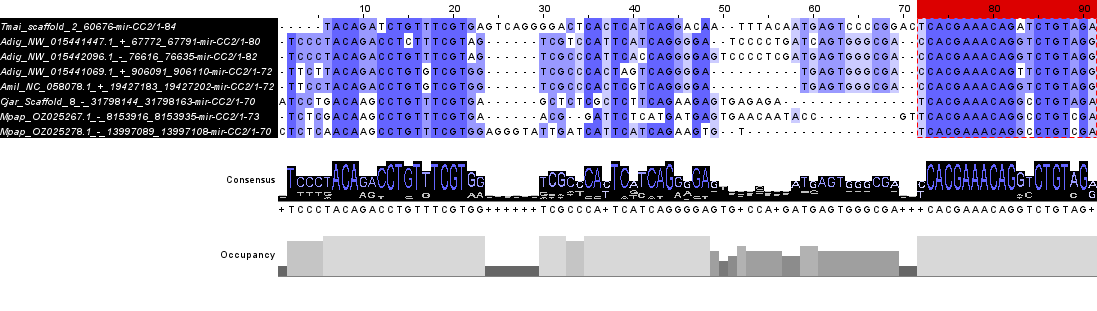


miR*-CC3*:


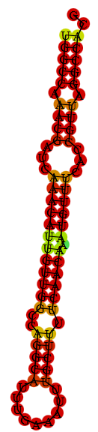
Ref: *Cqui*_JACTAT010000016.1_245715


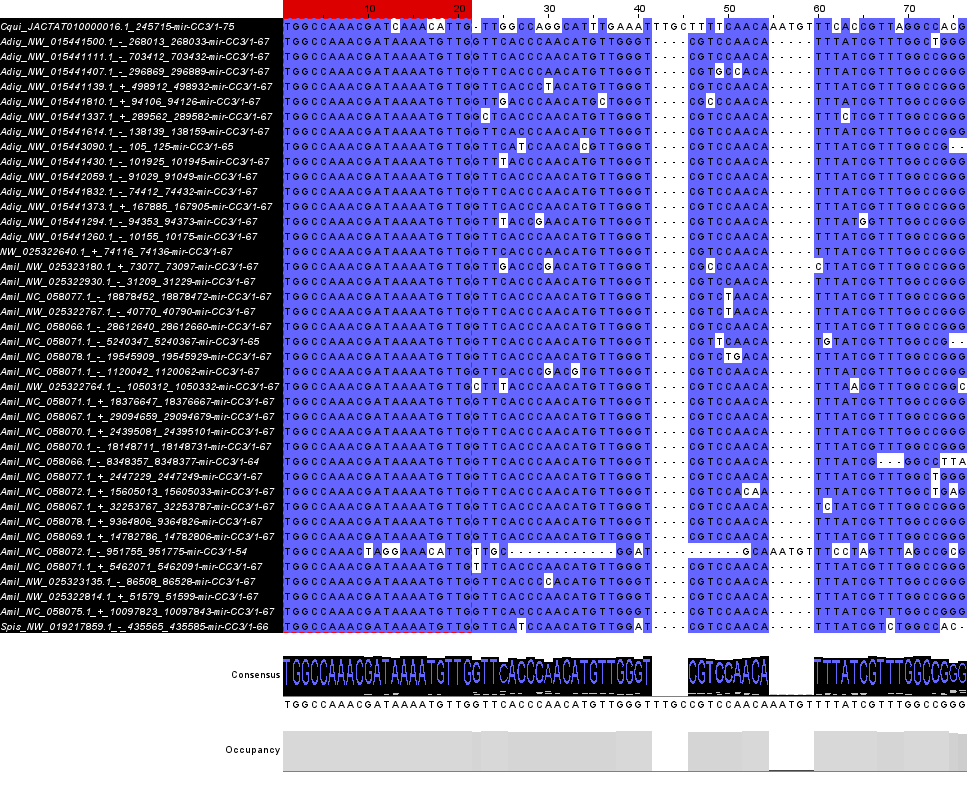


miR*-CC4*:


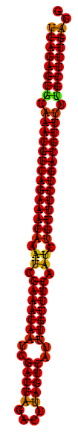
Ref: *Cqui*_JACTAT010000224.1_436035


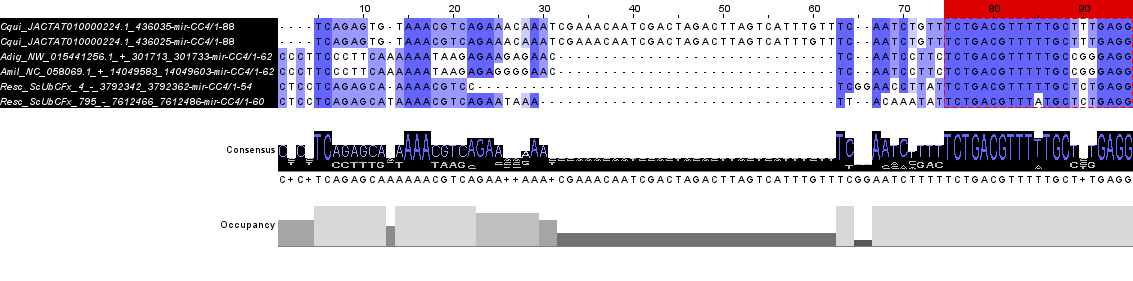


miR*-CC5*:


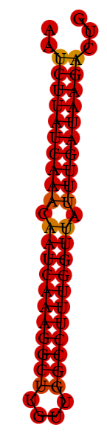
Ref: *Tmai*_scaffold_7_169664


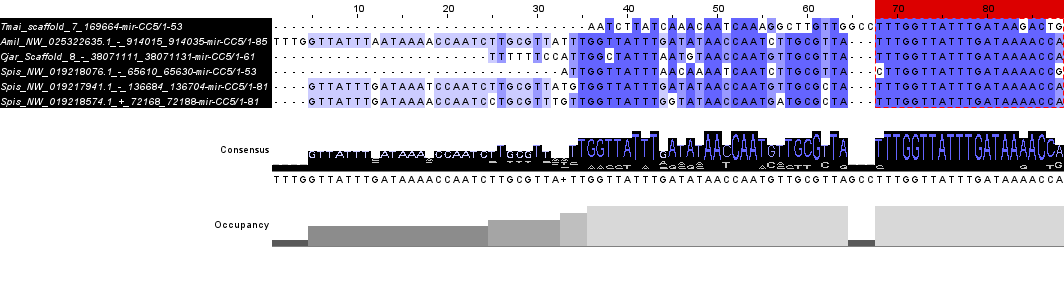


miR*-CC6*:


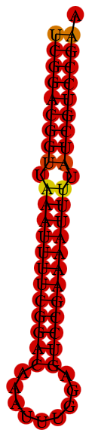
Ref: *Cqui*_JACTAT010000110.1_102516


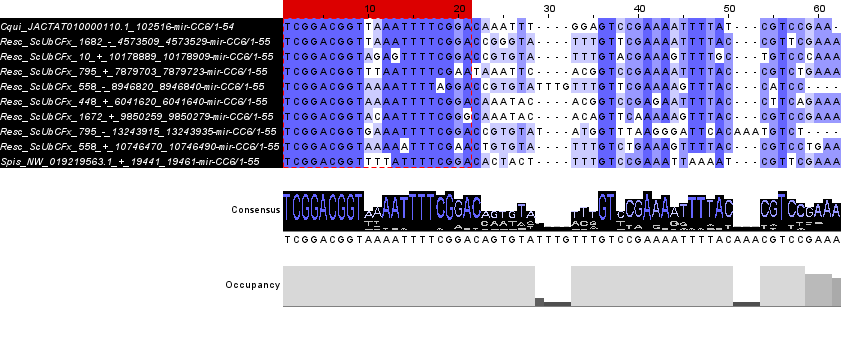


miR*-CC7*:


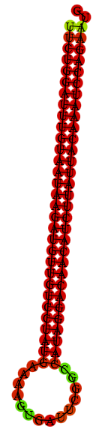
Ref: *Mpap*_OZ025277.1_622285


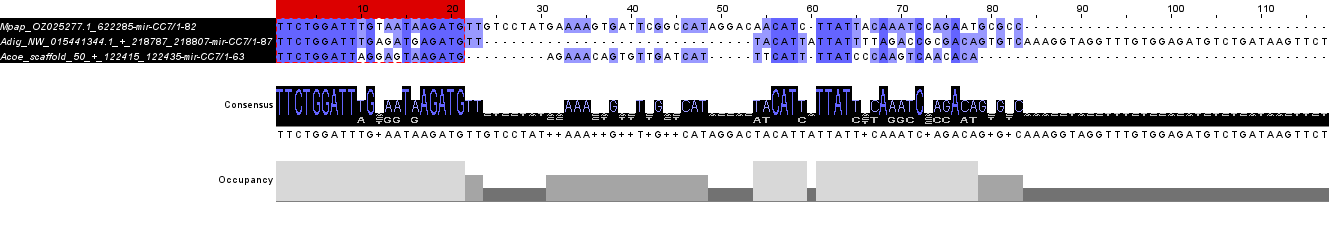


miR*-CC8*:


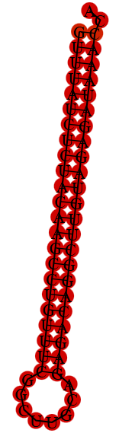
Ref: *Resc*_ScUbCFx_1672_889215


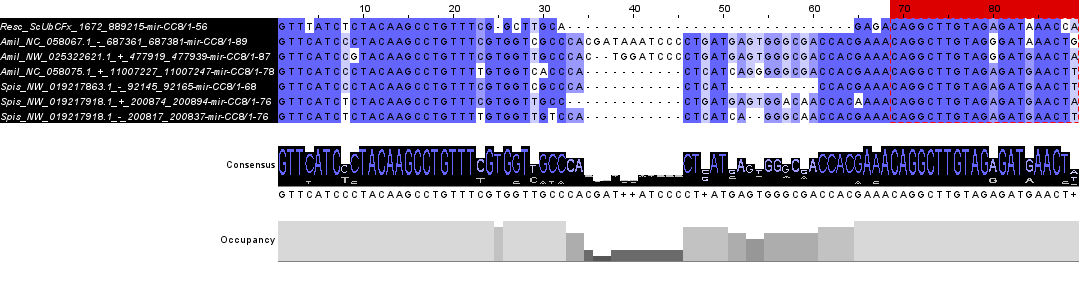


miR*-CC9*:


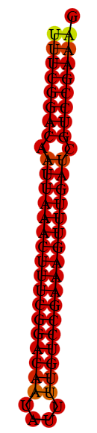
Ref: *Acoe*_scaffold_17_547614


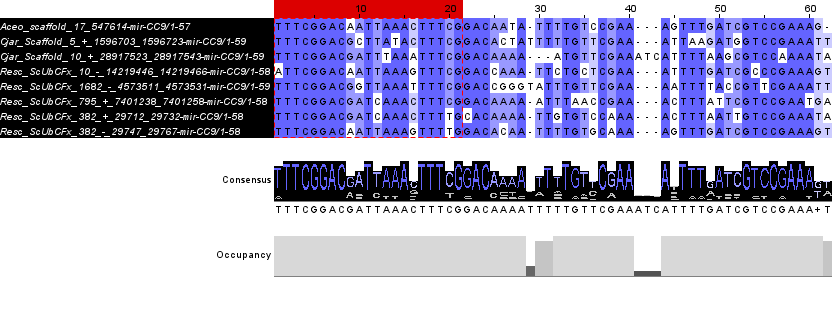


**Fig. S17.** Sequence alignments of conserved novel microRNAs in cubozoan, scyphozoans and anthozoans (miR*-CC1* to miR*-CC9*).

miR*-MC1*:


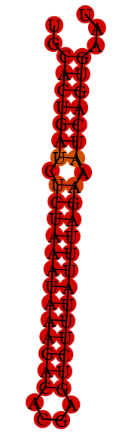
Ref: *Tmai*_scaffold_9_212333


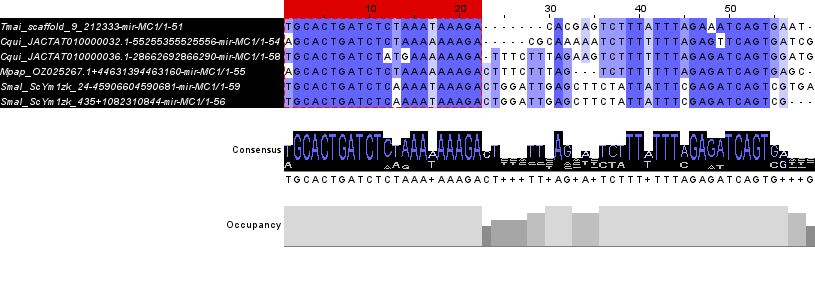


miR*-MC2*:


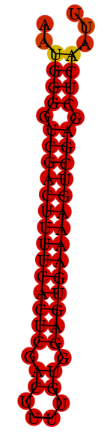
Ref: *Tmai*_scaffold_10_223052


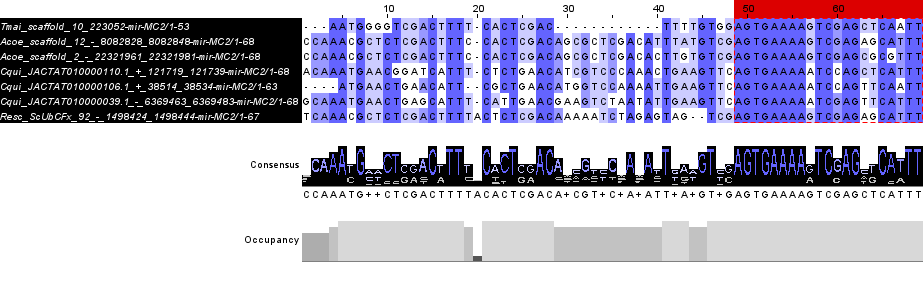


miR*-MC3*:


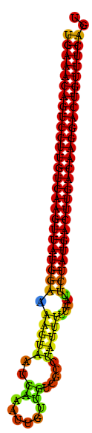
Ref: *Tmai*_scaffold_6_151844

*
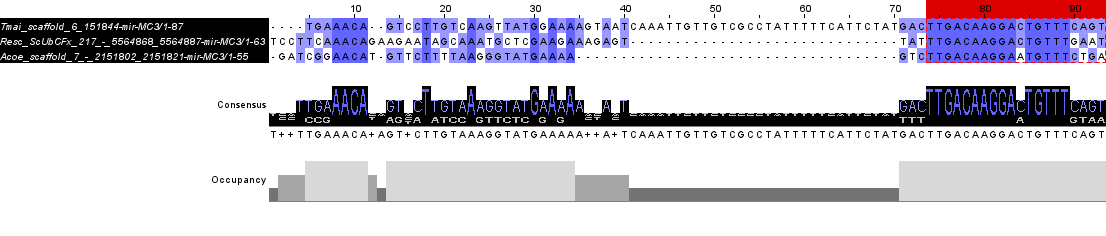
*

miR*-MC4*:


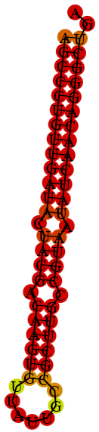
Ref: *Tmai*_scaffold_3_83965

*
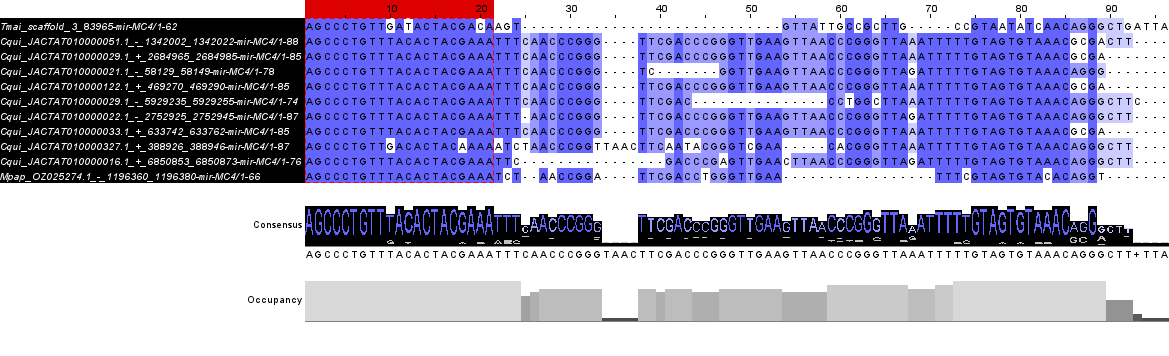
*

miR*-MC5*:


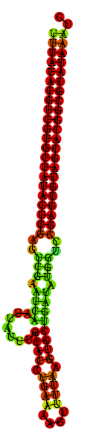
Ref: *Mpap_*OZ025272.1_283453

*
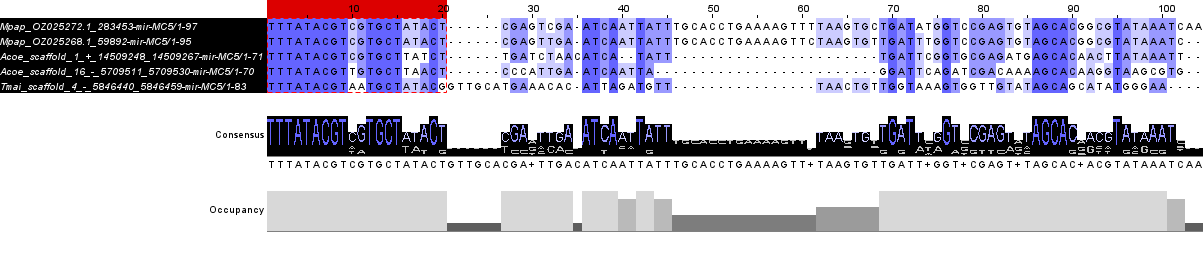
*

miR*-MC6*:

*
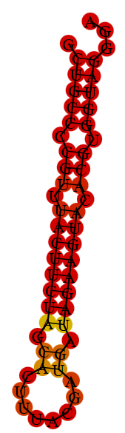
*Ref: *Resc_*ScUbCFx_10_122965

*
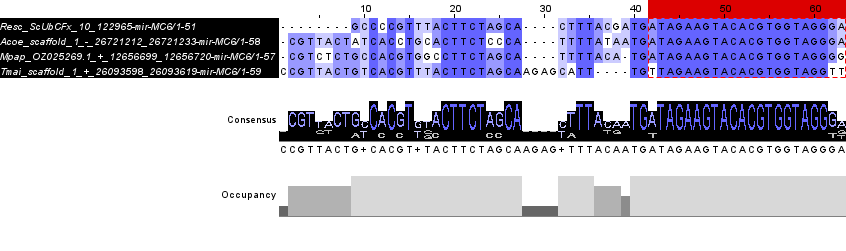
*

miR*-MC7*:

*
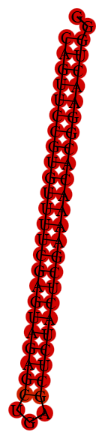
*Ref: *Cqui_*JACTAT010000031.1_478764


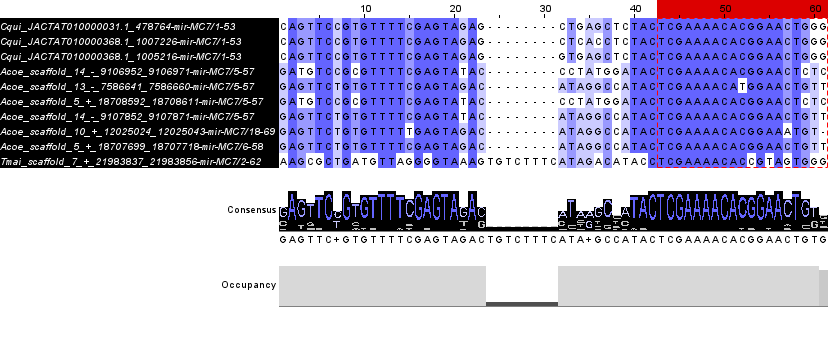


**Fig. S18.** Sequence alignments of conserved novel microRNAs in scyphozoans and cubozoan (miR*-MC1* to miR*-MC7*).

miR*-SC1*:


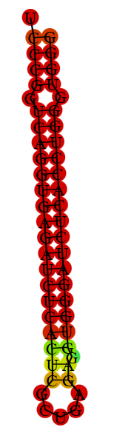
Ref: *Cqui_*JACTAT010000061.1_1254957


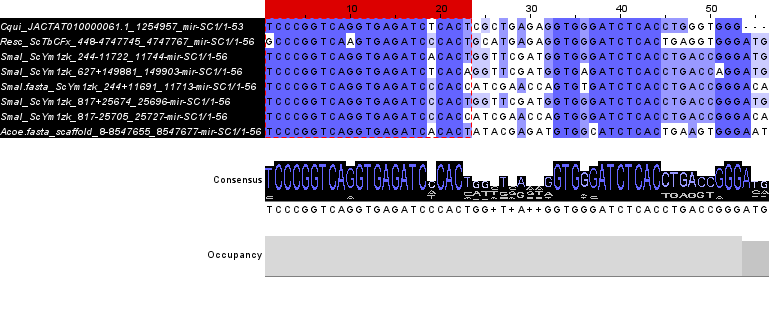


miR*-SC2*:

*
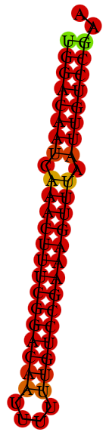
*Ref: *Acoe_*scaffold_9_351554


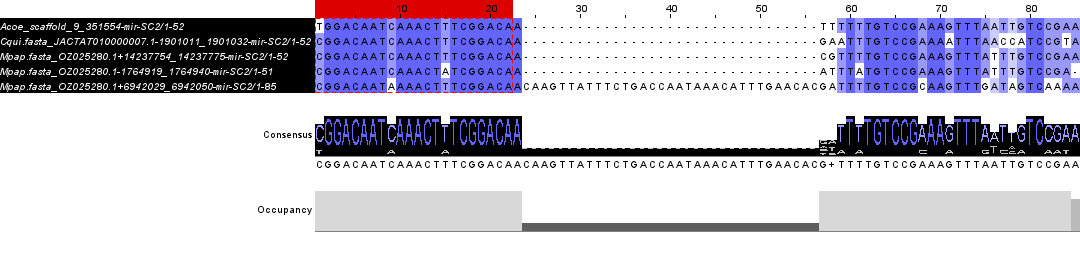


miR*-SC3*:


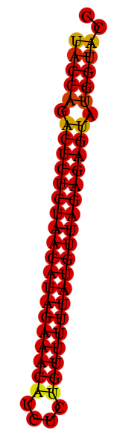
Ref: *Acoe_*scaffold_6_257069


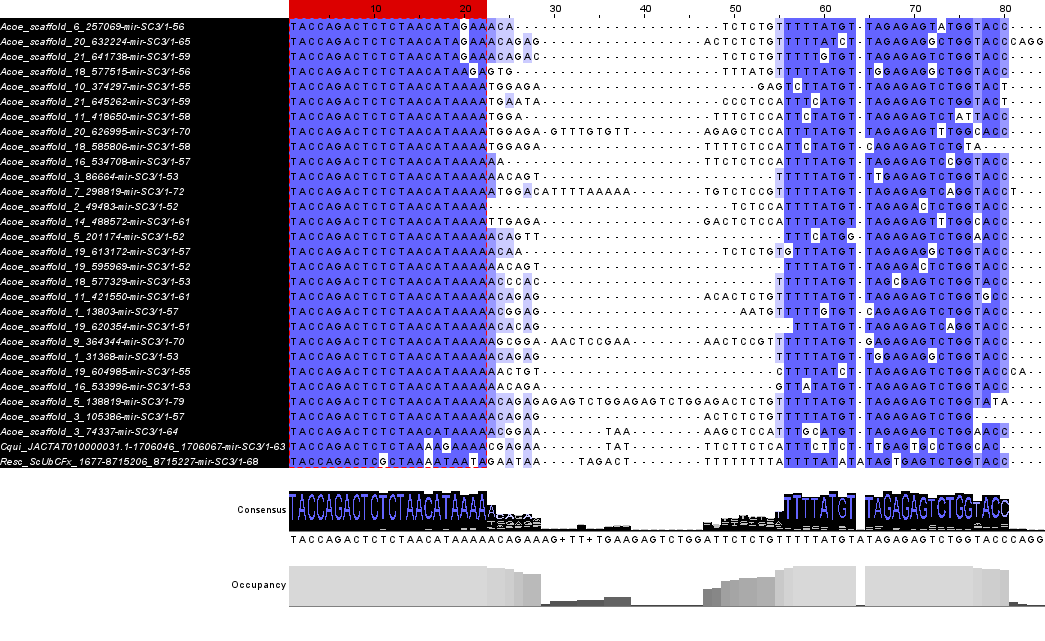


miR*-SC4*:


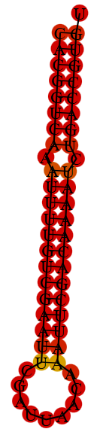
Ref: *Mpap_*OZ025282.1_894427


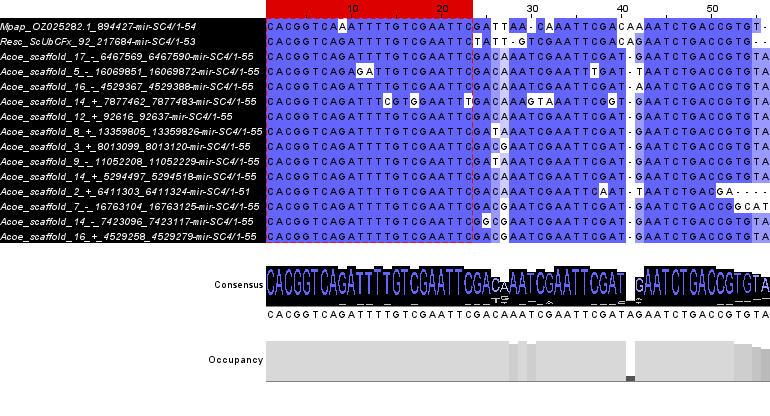


miR*-SC5*:


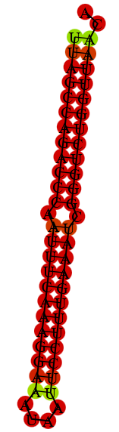
Ref: *Acoe_*scaffold_5_139005


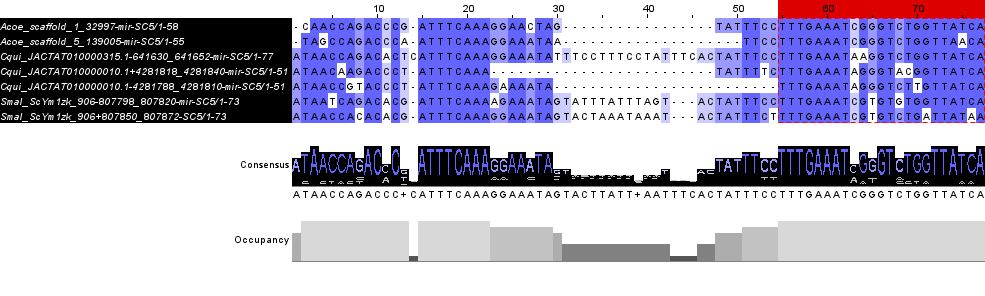


miR*-SC6*:

*
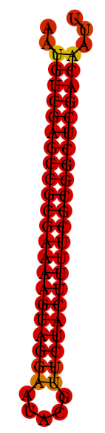
*Ref: *Acoe_*scaffold_11_417023


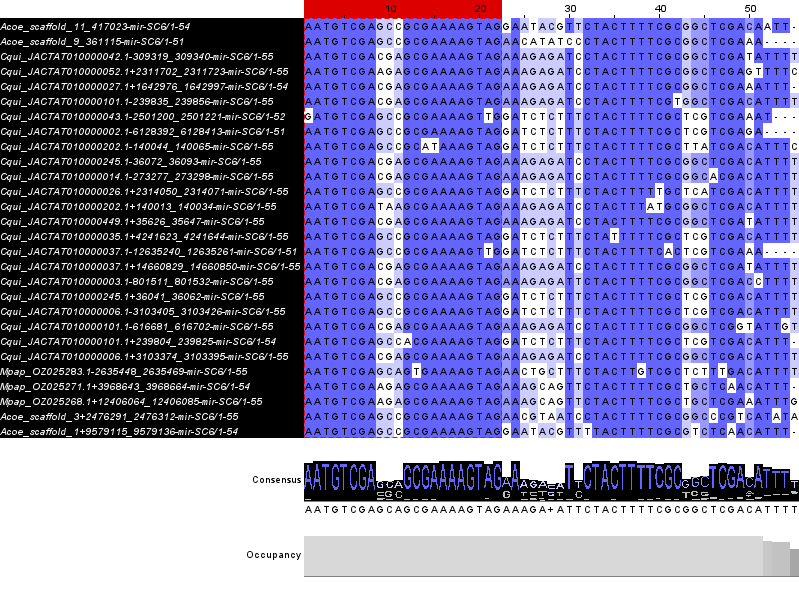


miR*-SC7*:


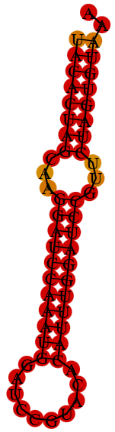
Ref: *Resc*_ScUbCFx_795_638234


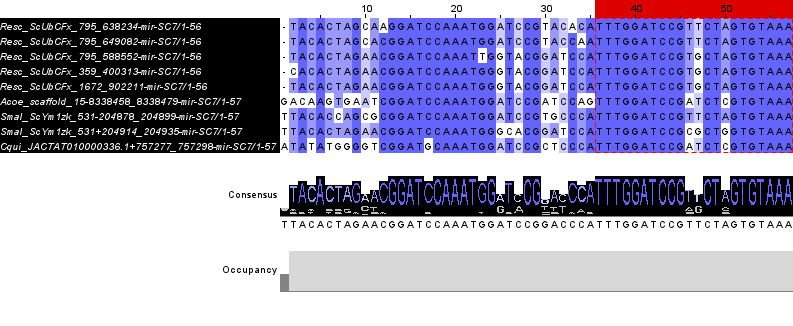


miR*-SC8*:

*
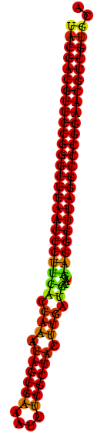
*Ref: *Cqui*_JACTAT010000040.1_851446


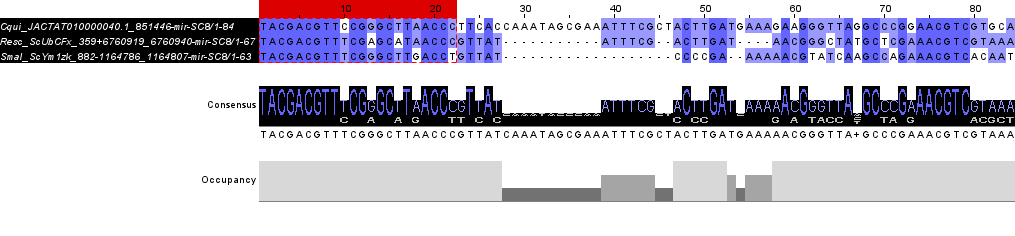


miR*-SC9*:

*
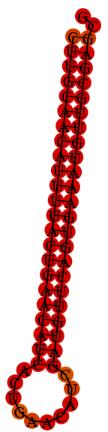
*Ref: *Smal*_ScYm1zk_618_21812


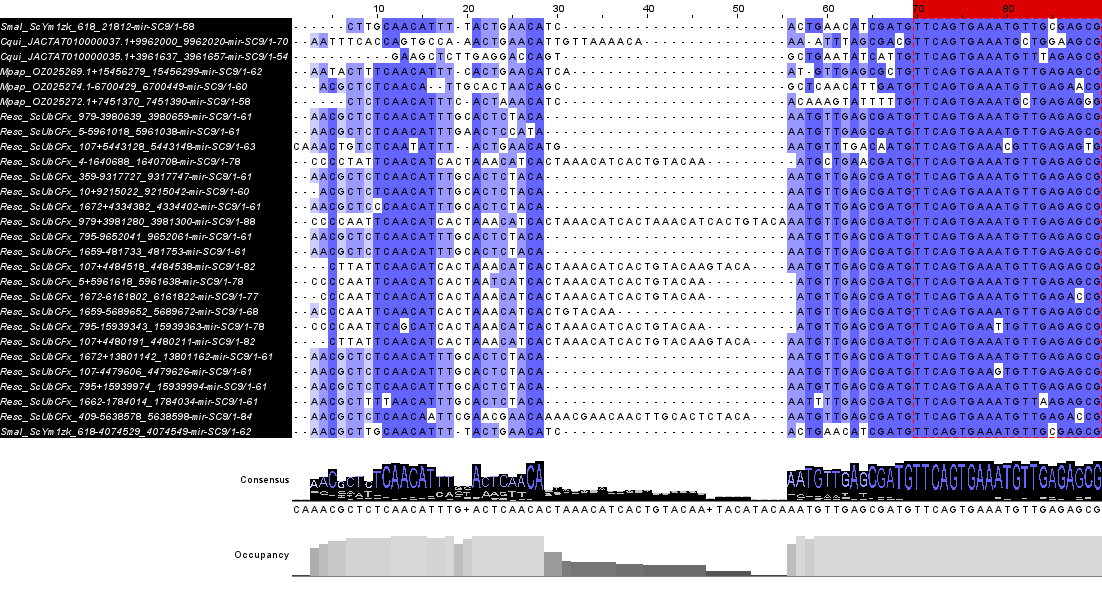


**Fig. S19.** Sequence alignments of conserved novel microRNAs in scyphozoans (miR*-SC1* to miR*-SC9*).


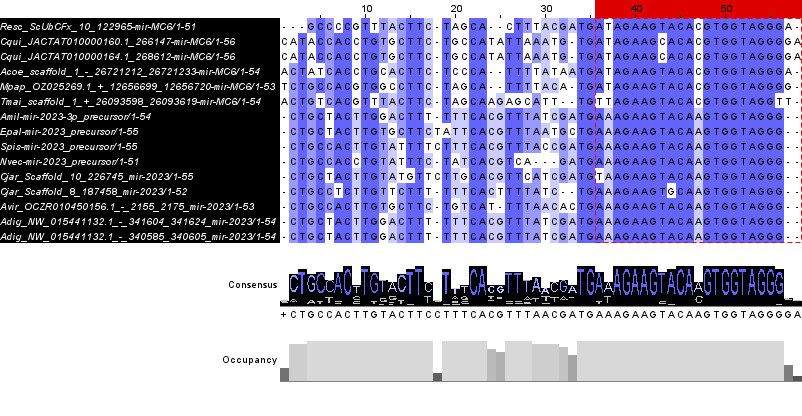


**Fig. S20.** Sequence alignments of miR-MC6 and miR-2023.
